# Liver frailty and impact of liver transplants on transplanted patients’ health

**DOI:** 10.1590/1518-8345.7330.4563

**Published:** 2025-05-19

**Authors:** Victor Fernandez-Alonso, Ana Maria Hernandez-Matias, Manuela Perez-Gomez, Leyre Rodriguez-Leal, Maria Nieves Moro-Tejedor

**Affiliations:** 1 Instituto de Investigación Sanitaria Gregorio Marañón, Madrid, Spain; 2 Universidad Autónoma de Madrid, Escuela Universitaria de Enfermeria de Cruz Roja, Madrid, Spain; 3 Hospital General Universitario Gregorio Marañon, Madrid, Spain

**Keywords:** Frailty, Liver Cirrhosis, Liver Transplantation, Alcoholics, Nursing, Rehabilitation

## Abstract

to analyse the Liver Frailty Index in a cohort of patients from their inclusion in the waiting list to one year after the liver transplant.

a cohort study with patients included in a liver transplant waiting list from January 2020 to December 2021. The variables were analysed and the hypothesis were contrasted by means of the Mann-Whitney U and Spearman’s correlation test, a paired measures test and multivariate analysis.

the sample consisted in n=51 patients with a mean age of 57.20 years old (SD=9.70), with 74.50% of men. The mean pre-transplant Liver Frailty index was 3.71 (SD=0.74), reaching higher values in patients with advanced liver disease (p=0.004), alcohol-related etiology (p=0.039) and unemployed (p=0.014). Liver frailty improved after the transplant (p<0.001), keeping a directly proportional correlation with age (p=0.014).

advanced liver disease, etiology related to alcohol and time in the waiting list exert impacts on liver frailty during the liver transplant process. Older liver transplanted patients are more frail.

## Highlights

**(1)** Frail patients are those whose etiology is related to alcohol. **(2)** Frail patients are those that are inactive or unemployed. **(3)** After the liver transplant, the patients’ physical and mental health improves. **(4)** Etiology, MELD-Na and time in the waiting list exert an impact on frailty. **(5)** Nurses should handle the management of cardiovascular risk factors.

## Introduction

Hepatic cirrhosis (HC) is the main diagnosis for including patients in a liver transplant (LT) waiting list in Spain^(1)^. The main cause of liver cirrhosis in Europe and Spain is alcoholic hepatitis or Alcohol-Related Liver Disease (ARLD), followed by Non-Alcoholic Steatohepatitis (NASH) and viral hepatitis B and C, whose prevalence has decreased in our setting due to the advances in its prevention and treatment^(2-3)^.

According to the National Transplant Organization (*Organización Nacional de Trasplantes*, ONT), 1,159 LTs were performed in Spain in 2022, which represents a rate of 24.4 for every 1 million inhabitants. A total of 1,294 with a mean age of 54.6 years old were included in the waiting list: 54% due to HC and 38% due to ARLD-induced cirrhosis. The time median in the waiting list was 55 days, with an Interquartile range (IQR) of 15-144 days, and mortality while in the waiting list was 2.4%^(1)^. LT continues to be the most effective treatment of choice for all types of liver failures, other etiologies not related to liver failure and liver cancer^(3)^.

Frailty is a term initially assessed from the Geriatrics point of view, and was defined as a “state of increased vulnerability when faced with physical stressors (surgery, for example) due to decreased physiological reserves”^(4)^. Various factors exert an influence of physical frailty, such as severity of the liver disease, age, muscle mass, nutritional state and comorbidities not related to the liver (diabetes, heart diseases, renal failure, etc.)^(4)^. This condition encompasses sarcopenia (skeletal muscle mass reduction), progressive immobility, energy expenditure reduction and malnutrition^(5)^. Physical frailty is a prevalent condition in cirrhotic patients and represents a clinical manifestation of muscle atrophy, malnutrition and functional decline^(6)^. There are several exams and/or tests to measure physical frailty in patients with liver disease and the Liver Frailty Index (LFI) stands out among them. It is a functional test based in the patient’s performance that is considered easy to apply and extremely precise^(7-8)^. Frailty and mortality of transplant candidates have been recently described in a review, with the result that liver frailty is a predictive value for mortality while in a waiting list^(9)^. The prevalence of liver frailty in LT candidates fluctuates between 15%^(10)^ and 21%^(11)^ and the one corresponding to frail patients is around 60%^(10-11)^. Liver frailty improves and its prevalence is reduced after an LT^(12)^. The liver frailty state is statistically related to admissions to Intensive Care units, longer hospitalisations and complications after the liver transplant, in addition to an increase in the economic costs^(11,13)^.

In the Digestive System Nursing advanced practice, nurses offer specialised care focused on the signs and symptoms associated with the cirrhotic stage, paying special attention to decompensated cirrhosis^(14)^. When a patient is accepted as a candidate for LT, the nurse conducts a comprehensive evaluation and develops a care plan in which the physical and mental health needs are considered, as well as the liver disease etiology and the Social Determinants of Health. This plan addresses the possible risks, both during the waiting period and after the LT.

The main objective of this study was to analyse the Liver Frailty Index in a cohort of patients, from their inclusion in the waiting list until one year after the LT, in addition to determining the impact exerted by an LT on the patients’ health.

## Method

### Study design

A longitudinal cohort study with an observational design was conducted, with two phases: one retrospective and the other prospective. *The Strengthening the Reporting of Observational studies in Epidemiology*(STROBE)^(15)^ guide for reporting results was used to report the study results.

### Context

The study was carried out in the LT unit from the Gregorio Marañón University General Hospital, a tertiary-level institution from the Community of Madrid, Spain. This hospital centre only performs LTs in adults and the mean number of LTs during the 2018-2021 period was 42.25^(1)^.

### Sample/Participants

The study population was comprised by all the adult patients included in the waiting list for a first elective LT in the LT unit of the Gregorio Marañón University General Hospital from Madrid, Spain, from January 2020 to December 2021. The patients that were selected once were accepted as candidates by the LT committee. In the consultation for evaluation and information about the transplant, they were informed regarding the study and provided the information; in addition, they signed an informed consent form.

The study sample was for convenience. No sample calculation was performed because we considered having the ability to access the entire sample of liver transplanted patients in the unit where the study was conducted. The patients included were all those that had received these type of transplant during that period. The patients that had been included for emergency LTs were also excluded due to their difficulty performing the physical exams and answering the questionnaires due to the advanced stage of their liver disease and to the presence of severe hepatic encephalopathy. Patients already subjected to another organ transplant were excluded, and those that required a second transplant with a time window over 1 month since the first LT were removed from the follow-up period. Finally, the patients that failed to finish the entire data collection during the study period were excluded.

### Variables

A number of sociodemographic variables were collected, namely: age, in years old; gender: male/female; marital status: single, with a partner-married; separated-divorced or widowed; schooling level: Elementary School, High School, Bachelor’s Degree, professional or university education; work situation: not working-unemployed, active or retiree-pensioner; and religion: professing some religion or not professing any religion^(16)^. Clinical variables associated with cardiovascular risk factors were also collected, namely: obesity, diabetes *mellitus* (DM), arterial hypertension, dyslipidaemia and smoking habit. The following variables related to the liver disease were collected at the patients’ inclusion in the waiting list: prognosis of the cirrhotic patient; Liver Frailty Index, anxiety/depression levels and etiology, determined by means of analytical-functional tests and hepatic serologies. The times in the waiting list (in days), hospital admission after the LT and readmission need during the first after the LT were collected. Twelve months after the LT, the clinical variables and those related to liver disease were reassessed.

### Data sources/Measures

Data collection was initiated after obtaining due approval from the Ethics Committee and the centre where the research was carried out, and having obtained the participants’ signed consent. At first, it was implemented retrospectively by accessing the records and medical histories of the patients included in the waiting list from January 2020 to February 2021. Subsequently, the LT consultation nurse prospectively collected the data in this consultation at the patient’s inclusion in the LT waiting list and, 12 months after the LT, the corresponding data collection was coordinated with the transplanted patients’ habitual follow-up visits. The research team was in charge of collecting the data from the patients’ medical histories.

In order to answer the questionnaires and calculate the Liver Frailty Index, clinical follow-up visits were coordinated with the study protocol. The questionnaires were handed in by the LT consultation nurse as each patient arrived and collected at their exit the same day. The Liver Frailty Index was calculated in the Nursing consultation, where the nurse led each patient in all three tests. Subsequently, the nurse filled in the index data.

### Indices and instruments

#### Liver Frailty Index (LFI)

All the patients in the LT waiting list underwent a physical frailty objective assessment while in in this list by using the Liver Frailty Index, which is a specific continuous index for patients with cirrhosis calculated from the scores obtained in three simple performance-based tests:

Dominant hand grip strength: the average value from three measurements using a hand dynamometer, in kilograms.

Standing up on a chair: how many seconds a patient takes to stand up five times on a chair with the arms crossed against their chest.

Balance test: the number of seconds (during a maximum of 10 seconds) that the patient can remain in each of the following three positions: standing up with the feet parallel to each other; semi-tandem, standing up with one foot slightly ahead the other; and tandem, standing up with one foot in front of the other tapping the front foot heel with the back foot toecap.

The online calculator available in http://liverfrailtyindex.ucsf.edu was used to calculate the Liver Frailty Index. The score obtained in the index categorized each patient as robust (<3.2), pre-frail (3.2-4.4) or frail (≥4.5)^(17)^. The Liver Frailty Index measurements were repeated at 12 months, coinciding with a visit for the post-transplant outpatient consultation. The frailty assessment that was closest to the transplant date was used as pre-transplant frailty measure in this study.

#### Model for End-Stage Liver Disease-Sodium (MELD-Na)

The Model for End-Stage Liver Disease-Sodium (MELD-Na) is a prognosis index that assesses the severity of cirrhosis. Its calculation is a logarithmic combination of bilirubin, INR, creatinine and serum sodium laboratory values. Values ≥15 indicate longer survival without LT^(18-19)^.

#### Hospital Anxiety and Depression Scale (HADS)

The Hospital Anxiety and Depression Scale (HADS) is one of the most used self-report instruments to detect emotional discomfort (anxiety and depression) in people with physical diseases^(20)^. It includes two subscales (HAS: Anxiety and HDS: Depression) with seven items that are scored by means of a 4-point Likert scale (from 0 to 3). The scores for each subscale can vary from 0 to 2. Values between 11 and 21 are considered as possible anxiety and/or depression cases^(20)^. This scale was validated for Spanish in the hospitalised population, having its internal consistency assessed by means of Cronbach’s alpha values of 0.80 for the full scale, 0.84 for the Depression subscale and 0.85 for the Anxiety one^(21)^. This tool has been employed at the international level for the psychological evaluation of patients with chronic liver disease^(22)^ and liver transplant recipients^(23)^.

### Biases

In order to avoid biases, the data collected retrospectively were gathered by the same researcher and extracted from each patient’s digital medical history. The data, questionnaires and tests collected at the Nursing consultation were also gathered by the same nurse in a systematised and rigorous way, thus avoiding observation and interpretation biases from other researchers.

### Data analysis

The data were stored in a database and analysed in the Statistical Package for the Social Sciences (SPSS), version 26.0 (SPSS Inc., Chicago, Illinois). An exploratory analysis was performed to identify outliers or extreme values and characterize the differences between the groups. The sociodemographic variables were recoded to ease the analysis without losing relevant information. Data distribution was studied with the Kolmogorov-Smirnov test. A descriptive data analysis and a comparison of the main hypothesis were performed using the Mann-Whitney U and Spearman’s correlation tests. The changes in LFI and MELD-Na were determined with a paired measures test. A multivariate analysis was performed through binary logistic regression to identify the variables that explained the pre- and post-transplant liver frailty behaviour. To ease interpretation, a dichotomous variables was created to classify the patients into two groups: frail or pre-frail against robust. The variables that showed a statistically significant relationship with frailty in the univariate analysis were included in this model. In all the cases, only p-values below 0.05 were considered as statistically significant.

### Ethical considerations

This study (as well as its written consent form) was approved by the Ethics Committee of the Gregorio Marañón University General Hospital (code IMPACT_TH from proceedings 02/2021) and was conducted according to the principles set forth in the Declaration of Helsinki^(24)^, as well as in Rule (EU) 2016/679 of the European Parliament and those from the Council for Data Protection dated April 27th, 2016^(25)^, thus ensuring proper data coding and pairing.

## Results

The study population was approximately n=86. A total of n=70 patients were included, of which n=11 (15.71%) and n=7 (10%) failed to finish the pre-transplant and post-transplant evaluations, respectively. In addition, one patient died after a massive stroke during the post-transplant follow-up period. Therefore, the total losses rose to 19 (27.14%) patients. The sample of patients analysed was n=51 and presented a mean age of 57.20 years old (SD=9.70), with n=38 (74.5%) men.

### Pre-transplant situation

As for the sociodemographic variables, 75.55% (n=37) had a partner; in turn, 13.7% (n=7) was single, 11.8% (n=6) separated and 2% (n=1) widowed. In all, 53.6% had schooling levels above mandatory education: 19.6% (n=10) Elementary School, 29.4% (n=15) High School, 19.6% (n=10) Bachelor’s Degree, 21.6% (n=11) professional training and 9.8% (n=5) university studies. A total of 35.3% (n=18) professed no religion, whereas 39.2% (n=20) were practising Catholics, 27.6% (n=9) were non-practising Catholics and 7.8% (n=4) professed other religions. Finally, 29.41% (n=15) had a job, whereas 35.3% (n=18) were unemployed and 35.3% (n=18) were retirees. At the time of inclusion in the waiting list, LFI shows statistical significance with work situation, being lower in those patients that remain active. As for the clinical variables and the cardiovascular risk factors at the patients’ inclusion in the waiting list, 35.29% (n=18) were diabetics, 37.25% (n=19) had high blood pressure, 27.45% (n=14) presented dyslipidaemia, 19.61% (n=10) were active smokers and 70.59% (n=36) were overweight or obese (Table 1).

**Table 1 -:** Bivariate analysis of the sociodemographic/clinical variables and the Liver Frailty Index before the transplant. Madrid, Spain, 2020-2021

Pre-LT* variable (n ^†^ =51)	Global n ^†^ (%)	LFI ^‡^ x̅ ^§^ (SD ^||^ )	Sig ^¶^ (p-value)
Gender			
Male	38 (74.5%)	3.72 (0.81)	0.897
Female	13 (25.5%)	3.67 (0.50)	
Marital status			
With a partner	37 (72.5%)	3.74 (0.66)	0.533
No partner	14 (27.5%)	3.62 (0.95)	
Schooling level			
High School or lower	25 (49%)	3.80 (0.59)	0.371
Higher Education	26 (51%)	3.62 (0.86)	
Work situation			
Active	36 (70.6%)	3.85 (0.69)	**0.014**
Not active	15 (29.4%)	3.67 (0.76)	
Religion			
Professes no religion	18 (35.3%)	3.80 (0.94)	0.315
Professes some religion	33 (64.7%)	3.66 (0.62)	
Diabetes *mellitus*			
Yes	18 (35.3%)	3.95 (0.59)	0.119
No	33 (64.7%)	3.52 (0.79)	
Arterial hypertension			
Yes	19 (37.3%)	3.74 (0.78)	0.876
No	32 (62.7%)	3.69 (0.73)	
Dyslipidaemia			
Yes	14 (27.5%)	4.02 (0.80)	0.069
No	37 (72.5%)	3.59 (0.69)	
Smoking habit			
Smoker	10 (19.6%)	3.66 (0.85)	0.822
Non-smoker	41 (80.4%)	3.72 (0.85)	
Etiology			
ARLD**	19 (37.3%)	3.98 (0.57)	**0.039**
Not ARLD**	32 (62.7%)	3.55 (0.79)	

*Pre-LT = Before liver transplant; ^†^n = Sample size; ^‡^LFI = Liver Frailty Index; ^§^x̅ = Mean value; ^||^SD = Standard Deviation; ^¶^Sig (p-value) = Mann-Whitney’s U statistical significance; **ARLD = Alcohol-Related Liver Disease

Severity of the liver disease according to the MELD-Na index reached a mean score of 15.04 (SD=6.36), presenting higher risk in 47.1% (n=24) of the patients. The mean LFI Score was 3.71 (SD=0.74), with the following results: 15.7% (n=8) frail, 66.7% (n=34) pre-frail and 17.6% (n=9) robust. Etiology of the liver disease was related to alcohol consumption in 37.25% (n=19) of the cases. The most prevalent LT indications by etiology were ARLD-induced cirrhosis with hepatocellular carcinoma (HCC) (15.7% [n=8]) and ARLD-induced cirrhosis (13.7% [n=7]). Among the LT indications due to non-ARLD etiologies, the most prevalent ones were cirrhosis induced by the hepatitis C virus and HACC (17.6% [n=9]), autoimmune hepatitis (9.8% [n=5]) and HCC alone (5.9% [n=3]).

As for the mental health sphere, the anxiety score recorded a median of 6 points [IQR 3-9], with 64.7% (n=33) for no anxiety, 15.7% (n=8) for anxiety risk and 19.6% (n=10) for anxiety. The depression score obtained a median of 4 [IQR 1-7], as follows: 80.4% (n=41) non-depressive state, 13.7% (n=7) depression risk and 5.9% (n=3) depressive state.

LFI was directly correlated with the MELD-Na score. In addition, it stood out that the HDS score was directly correlated with HAS and with the MELD-Na index and inversely correlated to the patients’ age (Table 2).

**Table 2 -:** Correlation between the quantitative variables and the Liver Frailty Index score before the transplant. Madrid, Spain, 2020-2021

LT* (n ^†^ **=51** )		**Age**	**BMI** ^‡^	**MELD-Na** ^§^	**HAS** ^||^	**HDS** ^¶^	LFI**
Age	r	1	0.058	-0.197	-0.208	**-0.323** ^††^	0.268
	p-value		0.686	0.166	0.142	**0.021**	0.057
BMI ^‡^	r	0.058	1	0.091	-0.123	0.071	0.030
	p-value	0.686		0.524	0.390	0.622	0.835
MELD-Na ^§^	r	-0.197	0.091	1	0.187	**0.315** ^††^	**0.392** ^‡‡^
	p-value	0.166	0.524		0.188	**0.024**	**0.004**
HAS ^||^	r	-0.208	-0.123	0.187	1	**0.570** ^‡‡^	-0.008
	p-value	0.142	0.390	0.188		**<0.001**	0.957
HDS ^¶^	r	**-0.323** ^††^	0.071	**0.315** ^††^	**0.570** ^‡‡^	1	0.098
	p-value	**0.021**	0.622	**0.024**	**<0.001**		0.495
LFI**	r	0.268	0.30	**0.391** ^‡‡^	-0.008	0.098	1
	p-value	0.057	0.835	**0.004**	0.957	0.495	

*LT = Liver Transplant; ^†^n = Sample size; ^‡^BMI = Body Mass Index; ^§^MELD-Na = Model for End-Stage Liver Disease-Sodium; ^||^HAS = Hospital Anxiety Scale; ^¶^HDS = Hospital Depression Scale; **LFI = Liver Frailty Index; ^††^The correlation is significant at the 0.05 level (bilateral); ^‡‡^The correlation is significant at the 0.01 level (bilateral)

### Post-transplant situation

All 51 patients were elective liver transplant recipients. After the LT, n=3 (5.88%) required an emergency second transplant due to primary graft malfunction. The mean LFI score after the LT was 3.39 (SD=0.77), with n=2 (3.9%) frail patients, n=32 (62.7%) pre-frail ones and n=17 (33.3%) robust subjects (Table 3), observing a statistically significant change (p<0.001). Thus, n=15 (29.41%) improve in terms of frailty, n=33 (64.71%) remain unchanged and frailty worsens when compared to the pre-transplant situation in n=3 (5.88%) cases.

**Table 3 -:** Impact of the liver transplant, before the surgery and at 1 year. Madrid, Spain, 2020-2021

	Pre-LT* (%, SD ^‡^ )	Post-LT ^†^ (%, SD ^‡^ )	Statistical value	Sig ^§^ (p-value)
Diabetes *mellitus*				
Yes	18 (35.29%)	29 (56.86%)	-3,065	**0.007** ^||^
No	33 (64.71%)	22 (43.14%)		
Arterial hypertension				
Yes	19 (37.25%)	33 (64.71%)	-3.253	**0.004** ^||^
No	32 (62.75%)	18 (35.29%)		
Dyslipidaemia				
Yes	14 (27.45%)	16 (31.37%)	-0,496	0.804
No	37 (72.55%)	35 (68.63%)		
Smoker				
Yes	10 (19.6%)	5 (9.8%)	1.940	0.058
No	41 (80.4%)	46 (90.2%)		
LFI ^¶^	3.71 (SD 0.74)	3.39 (SD 0.77)	3.764	<0.001**
MELD-Na ^††^	15.04 (SD 6.36)	9.05 (SD 3.07)	6.041	<0.001**
BMI ^‡‡^	28.07 (SD 4.91)	26.82 (SD 5.57)	2.170	0.035**
HAS ^§§^	6.49 (SD 3.82)	4.94 (SD 3.67)	3.257	0.002**
HDS ^||||^	4.43 (SD 3.59)	3.14 (SD 3.58)	2.520	0.015**

*Pre-LT = Before the liver transplant; ^†^Post-LT = After the liver transplant; ^‡^SD = Standard Deviation; ^§^Sig (p-value) = Statistical significance; ^||^Student’s t; ^¶^LFI = Liver Frailty Index; **Chi-square; ^††^MELD-Na = Model for End-Stage Liver Disease-Sodium; ^‡‡^BMI = Body Mass Index; ^§§^HAS = Hospital Anxiety Scale; ^||||^HDS = Hospital Depression Scale

The median of days in the transplant waiting list was 114 [IQR 46-202] and the median of days corresponding to hospital admission for the transplant process was 16 [IQR 13-22]. No statistically significant differences were recorded between the LFI score and time in the waiting list (p=0.124) or in the hospital admission time (p=0.630). Although the differences were not statistically significant, our results describe that frail patients tend to require longer hospitalisations than those in the pre-frail and robust states.

During the first year after the LT, 56.9% (n=29) was hospitalized at least once. The LFI score was higher among those that had to hospitalized (3.73 [SD=0.72]) when compared to the subjects not requiring hospitalization (3.68 [SD=0.78]) because the p-value was above 0.05. In relation to the cardiovascular risk factors, there was increase in diabetes and arterial hypertension. On the other hand, a statistically significant improvement was observed in the Body Mass Index, the Liver Frailty Index and the Anxiety and Depression scores (Table 3). In addition, regarding the LFI score one year after the LT, we found a statistically significant correlation with the MELD-Na index and age (Table 4), but we did not notice any significant relationship with the clinical variables. As for the differences in the LFI values, the mean pre-transplant score was 3.71 (SD=0.74) against the post-transplant one, which was 3.39 (SD=0.77) and, in the case of the pre-transplant MELD-Na value (15.04 [SD=6.36]) against the post-transplant score, which was 9.06 (SD=3.0), representing statistically significant differences (p<0.001).

**Table 4 -:** Correlation between the quantitative variables and the Liver Frailty Index score after the transplant. Madrid, Spain, 2020-2021

LT* **(n** ^†^ **=51)**		**Age**	**BMI** ^‡^	**MELD-Na** ^§^	**HAS** ^||^	**HDS** ^¶^	LFI**
Age	r	1	-0.098	0.232	**-0.367** ^††^	-0.221	**0.343** ^‡‡^
	p-value		0.495	0.101	**0.008**	0.119	**0.014**
BMI ^‡^	r	-0.098	1	-0.044	-0.061	0.068	-0.127
	p-value	0.495		0.761	0.672	0.636	0.373
MELD-Na ^§^	r	0.232	-0.044	1	-0.117	-0.260	**0.287** ^‡‡^
	p-value	0.101	0.761		0.414	0.065	**0.041**
HAS ^||^	r	**-0.367** ^††^	-0.061	-0.117	1	**0.528** ^††^	-0.136
	p-value	**0.008**	0.672	0.414		**<0.001**	0.341
HDS ^¶^	r	-0.221	0.068	-0.260	**0.528** ^††^	1	0.146
	p-value	0.119	0.636	0.065	**<0.001**		0.308
LFI**	r	**0.343** ^‡‡^	-0.127	**0.287** ^‡‡^	-0.136	0.146	1
	p-value	**0.014**	0.373	**0.041**	0.341	0.308	

*LT = Liver Transplant; ^†^n = Sample size; ^‡^BMI = Body Mass Index; ^§^MELD-Na = Model for End-Stage Liver Disease-Sodium; ^||^HAS = Hospital Anxiety Scale; ^¶^HDS = Hospital Depression Scale; **LFI = Liver Frailty Index; ^††^The correlation is significant at the 0.01 level (bilateral); ^‡‡^The correlation is significant at the 0.05 level (bilateral)

### Multivariate analysis

In the multivariate regression analysis after adjusting the covariates associated with liver frailty, the model at the patients’ inclusion in the liver transplant waiting list presented a pseudo R² of 0.3268, which indicates good fit of the model. Assessed by means of the Area Under the ROC Curve (AUC), the discriminant capacity was 0.8677, showing high precision in differentiating between the pre-frail/frail and robust groups. In addition, the Hosmer-Lemeshow goodness-of-fit test was not significant [χ²(8)=4.29; *p*=0.8301], which confirms that the models suitably fits the data observed. In this model, the MELD-Na index was significantly associated with frailty (OR=1.309; 95% CI: 1-05–1-63; *p*=0-017), as was the case with time in the liver transplant waiting list (OR=1.010; 95% CI: 1.00–1.02; *p*=0.042); however, the etiology of the disease related to alcohol consumption (OR=4.841; 95% CI: 0.48–48.83) did not reach statistical significance (*p*=0.181). One year after the liver transplant, the model showed a pseudo-R² of 0.1598, reflecting moderate fit, with AUC of 0.7630 that indicates acceptable discriminant ability. The Hosmer-Lemeshow was not statistically significant [χ²(8)=8.12; *p*=0.4220], thus supporting validity of the model; the MELD-Na index showed an association that was close to statistical significance (OR=1.237; 95% CI: 0.97–1.58; *p*=0.091), as was the case with time in the waiting list (OR=1.006; 95% CI: 1.00–1.01; *p*=0.064), with the etiology related to alcohol consumption as the variable that presented a statistically significant association with liver frailty (OR=4.694; 95% CI: 1.05–20.94; *p*=0.043) (Table 5).

**Table 5 -:** Variables that exert an influence on liver frailty at inclusion in the transplant waiting list and one year after the liver transplant. Multivariate analysis. Madrid, Spain, 2020-2021

**Variable**	**Inclusion in liver transplant waiting list**						**One year after liver transplant**			
	**Regression Coefficient (β)**	**Standard Error**	z*	Odds Ratio (95% Confidence Interval)	**Sig** ^†^ **(p-value)**	**Regression Coefficient (β)**	**Standard Error**	z*	**Odds Ratio (95% Confidence Interval)**	**Sig** ^†^ **(p-value)**
MELD-Na ^‡^	0.270	0.113	2.39	1.309 (1.050 – 1.633)	**0.017**	0.213	0.156	1.69	1.237 (0.966 – 1.584)	**0.043**
Time in waiting list (days)	0.011	0.005	2.03	1.010 (1.000 – 1.021)	**0.042**	0.007	0.003	1.85	1.006 (0.999 – 1.013)	0.064
ARLD ^§^	1.577	1.179	1.34	4.841 (0.480 – 48.832)	0.181	1.564	3.581	2.03	4.694 (1.052 – 20.941)	0.091
Constant	-3.634	1.673	-2.17	0.026 (0.001 – 0.701)	0.030	-2.518	0.110	-1.84	0.080 (0.005 – 1.183)	0.066

*z = Logistic regression statistical value; ^†^Sig (p value) = Statistical significance; ^‡^MELD-Na = Model for End-Stage Liver Disease-Sodium; ^§^ARLD = Alcohol-Related Liver Disease

## Discussion

The most frail patients are those with advanced decompensated hepatic function, indicated by a high MELD-Na index. The patients with alcohol-related liver disease and those who are unemployed or inactive are also more frail. One year after the LT, frailty remains significantly related to the MELD-Na index and to age, with older patients suffering it the most.

LFI supports MELD-Na in predicting mortality among patients with liver dysfunction^(26)^. In our results, LFI maintains that correlation in a direct and significant way, both before and after the transplant. At inclusion in the waiting list, higher LFI values are also significantly related to ARLD. Alcohol consumption and the metabolic syndrome worsen sarcopenia, exerting negative effects on muscles and growth signalling, and increasing insulin resistance^(27)^. Hepatology nurses should educate the population regarding the consequences of this problem on liver disease, advising on its effects and preventing consumption and abusive consumption^(28)^.

The relationship between live frailty, LFI, time until the transplant and alcohol-induced cirrhosis in transplanted patients is complex and significant. The patients with alcohol-induced cirrhosis usually present higher liver frailty degrees, which can exert an influence on the waiting time to be transplanted, with possible worsening of their overall health state and of the LT-associated risk. Constant evaluation and adequate management of the LFI index are essential to improve the outcomes in transplanted patients with a background of alcohol-related liver disease.

Our results reinforce the implementation of the 2030 Agenda Sustainable Development Goals, with *Ensure healthy lives and promote well-being for all at all ages* as the main goal and, in particular, *Strengthen the prevention and treatment of substance abuse, including narcotic drug abuse and harmful use of alcohol* ^(29)^. Although our results describe the alcohol-related etiology as more frail and our sample was limited, the proposal set for in the study conclusion is maintained: although frailty is more common in patients with ARLD, it is associated with mortality in the waiting list regardless of the cirrhosis etiology and should be applied in all etiologies of this pathology^(30)^.

Inactive patients (either due to unemployment or to retirement) are more frail. Liver failure deteriorates a person’s physical and cognitive states and worsens the physical conditions required to perform a given job^(13)^. It is known that frailty is related to sarcopenia, malnutrition and inactivity; although it can be a consequence, it can also be an aggravating factor^(31)^, reason why we should focus on inactivity as a risk for increased frailty. The literature describes the importance of conditioning or pre-conditioning programs in cirrhotic patients that are transplant candidates^(32-35)^. For these programs, nurses are profiled as the reference professionals to assess frailty, as well as for direct and close collaboration with Primary Health Care professionals and the patients’ relatives and their environment, taking into account their socioeconomic and cultural levels^(7)^. Nursing care specialised in cirrhosis or Hepatology is focused on nutrition, on educating patients and caregivers about possible complications, on awareness regarding the risk of falls and skin frailty care, and on maintaining periodic follow-up and reviewing drug compliance^(28)^. The American Society of Transplantation proposed a multidimensional frailty assessment in the everyday practice that included four measures: Karnofsky index, Activities of Daily Living (ADLs), Liver Frailty Index and six-minute gait test^(36)^. The interventions to treat liver frailty should focus on physical exercise, nutrition, pharmacological treatment and cognitive training^(37)^.

After the LT, the more frail patients are at a higher risk of complications and longer hospitalizations^(11,38)^. Our findings show no significant differences between the frailty groups, but a similar trend in the results^(11)^. One year after the LT, LFI is only directly related to age, returning to its primary concept from Geriatrics. Our results also show that liver frailty continues to be affected by ARLD etiology, MELD-Na and time in the waiting list one year after the LT.

As described in the scientific literature, LTs improve the patients’ life and health^(39-40)^. Our results describe improvements in the physical and mental dimensions, in consonance with those obtained in other studies. Nevertheless, we should pay attention to worsening of the cardiovascular risk factors^(39)^. In line with the medical treatment, which is focused on achieving the minimum immunodepression dose that will allow viability of the transplanted graft preventing metabolic syndrome^(41)^, renal dysfunction^(42)^ and cancer^(43)^, from the Nursing area we should focus on controlling and managing the symptoms based on modifiable health habits, diet, physical exercise and preventing the consumption of toxic substances such as tobacco and alcohol, maintaining adherence to the immunosuppressant treatment^(44)^.

Among the secondary findings of our study, we should note that depression is directly linked to anxiety and MELD-Na during the waiting list period, describing that the patients with more advanced liver disease and weakened physical health also suffer effects on their mental health. These results have already been described, concluding in the need to assess depression in frail patients^(37,45)^. Our results are indirectly correlated with age, describing that, at inclusion in the waiting list, younger patients present higher depression scores. One year after the LT, it is the anxiety score that is indirectly correlated with age. Our results point out that the mental health sphere is more affected in younger patients. This impact can be due to the fears and complications inherent to having to undergo an LT at a young age, as well as to the consequences in their personal life, work- and social-related impacts and possible recurrence of the liver disease^(46-48)^.

Nurses should focus their interventions on preventing alcoholism and on educating the population regarding its consequences for health. They should also lead physical conditioning or pre-conditioning programs, fostering physical exercise targeted at frail patients to improve their quality of life and prevent complications after the transplant. The patients’ mental health should be assessed during the entire process, focusing on the youngest patients. Nursing care for transplanted patients should be targeted at managing the cardiovascular risk factors based on changing habits such as diet and physical exercise and on preventing the consumption of toxic substances such as tobacco and alcohol.

We can mention the following as a strength of our study: having carried out the prospective follow-up using indices and instruments of recent implementation in LT units. The main limitations would be the sample size, the fact that the study was developed in a single centre, the losses in finishing data collection, and the exclusion of patients included as emergency transplants. In addition, no sociodemographic data were collected one year after the LT, which may have limited the analysis of the results.

## Conclusion

The Liver Frailty Index describes the physical impact exerted by liver disease and improves after an LT. The patients with advanced liver disease, alcohol-related etiology and categorized as unemployed are more frail during the pre-transplant period. The cardiovascular, diabetes and arterial hypertension risk factors worsen after an LT. The patients’ mental health and frailty levels improve after the LT, and the oldest subjects are the most frail. Alcohol-related etiology, MELD-Na and time in the waiting list exert impacts on frailty during the entire process.

## References

[1] Ministerio de Sanidade (ES); (2023). Organización Nacional de Trasplantes. Donation and transplant activity [Internet].

[2] Bataller R., Cabezas J., Aller R., Ventura-Cots M., Abad J., Albillos A. (2019). Alcohol-related liver disease. Clinical practice guidelines. Consensus document sponsored by AEEH. Gastroenterol Hepatol.

[3] Millson C., Considine A., Cramp M. E., Holt A., Hubscher S., Hutchinson J. (2020). Adult liver transplantation: A UK clinical guideline - part 1: pre-operation. Frontline Gastroenterol.

[4] Lai J. C., Feng S., Terrault N. A., Lizaola B., Hayssen H., Covinsky K. (2014). Frailty Predicts Waitlist Mortality in Liver Transplant Candidates. Am J Transplant.

[5] Lai J. C., Tandon P., Bernal W., Tapper E. B., Ekong U., Dasarathy S. (2021). Malnutrition, Frailty, and Sarcopenia in Patients With Cirrhosis: 2021 Practice Guidance by the American Association for the Study of Liver Diseases. Hepatology.

[6] Puchades Renau L, Herreras López J, Cebrià i Iranzo MÀ, Cezón Serrano N, Di Maira T, M Berenguer (2021). Frailty and Sarcopenia in Acute-on-Chronic Liver Failure. Hepatol Commun.

[7] Puchades Renau L, Herreras-López J, Cebrià i Iranzo M Àngels, Cezón Serrano N, Berenguer Haym M (2021). Physical frailty in liver transplantation. Rev Española Enfermedades Dig.

[8] Jutras G., Lai J. C. (2024). The Liver Frailty Index: a model for establishing organ-specific frailty metrics across all solid organ transplantation. Curr Opin Organ Transplant.

[9] Moosavi S. A., Mashhadiagha A., Taherifard E., Fallahzadeh M. A., Motazedian N., Sayadi M. (2023). Frailty as a predictor of poor outcomes among patients awaiting liver transplant: a systematic review and meta-analysis. Gastroenterol Hepatol Bed Bench.

[10] Thuluvath A. J., Duarte-Rojo A., Lai J. C., Peipert J., Dietch Z. C., Siddiqui O. (2024). Brief PROMIS Assessment Screens for Frailty and Predicts Hospitalizations in Liver Transplant Candidates. Transplantation.

[11] Puchades L., Herreras J., Ibañez A., Reyes E., Crespo G., Rodríguez-Perálvarez M. (2023). Waiting time dictates impact of frailty: a spanish multicentre prospective study. JHEP Rep.

[12] Lai J. C., Shui A. M., Duarte-Rojo A., Rahimi R. S., Ganger D. R., Verna E. C. (2023). Association of Frailty With Health-Related Quality of Life in Liver Transplant Recipients. JAMA Surg.

[13] Tandon P., Zanetto A., Piano S., Heimbach J. K., Dasarathy S. (2023). Liver transplantation in the patient with physical frailty. J Hepatol.

[14] Fabrellas N., Carol M., Palacio E., Aban M., Lanzillotti T., Nicolao G. (2020). Nursing Care of Patients With Cirrhosis: The LiverHope Nursing Project. Hepatology.

[15] Cuschieri S. (2019). The STROBE guidelines. Saudi J Anaesth.

[16] Paglione H. B., Oliveira P. C., Mucci S., Roza B. A., Schirmer J. (2019). Quality of life, religiosity, and anxiety and depressive symptoms in liver transplantation candidates. Rev Esc Enferm USP.

[17] Tejedor M., Selzner N., Berenguer M. (2022). Are MELD and MELDNa Still Reliable Tools to Predict Mortality on the Liver Transplant Waiting List?. Transplantation.

[18] Ruf A., Dirchwolf M., Freeman R. B. (2022). From Child-Pugh to MELD score and beyond: Taking a walk down memory lane. Ann Hepatol.

[19] Pardo F., Pons J. A., Castells L., Colmenero J., Gómez M. A., Lladó L. (2018). VI consensus document by the Spanish Liver Transplantation Society. Gastroenterol Hepatol.

[20] Zigmond A. S., Snaith R. P. (1983). The Hospital Anxiety and Depression Scale. Acta Psychiatr Scand.

[21] Herrero M. J., Blanch J., Peri J. M., Pablo J., Pintor L., Bulbena A. (2003). A validation study of the hospital anxiety and depression scale (HADS) in a Spanish population. Gen Hosp Psychiatry.

[22] Whitsett M. P., Banerjee A. G., Serper M. (2022). Assessment of mental health in patients with chronic liver disease. Clin Liver Dis.

[23] Sözen K. K., Karabulut N. (2023). Determination of the Relationship Between Family and Social Support and Anxiety-Depression Levels in Liver Transplant Patients. Clin Exp Health Sci.

[24] World Medical Association. (2013). World Medical Association Declaration of Helsinki: Ethical principles for medical research involving human subjects. JAMA.

[25] Reglamento (UE) (2016). Reglamento (UE) 2016/679 del Parlamento Europeo y del Consejo, de 27 de abril de 2016, relativo a la protección de las personas físicas en lo que respecta al tratamiento de datos personales y a la libre circulación de estos datos y por el que se deroga la Directiva 95/46/CE (Reglamento general de protección de datos).

[26] Lai J. C., Covinsky K. E., Dodge J. L., Boscardin W. J., Segev D. L., Roberts J. P. (2017). Development of a novel frailty index to predict mortality in patients with end-stage liver disease. Hepatology.

[27] Redman J. S., Kaspar M., Puri P. (2022). Implications of pre-transplant sarcopenia and frailty in patients with non-alcoholic steatohepatitis and alcoholic liver disease. Transl Gastroenterol Hepatol.

[28] Garcia-Pagan J. C., Francoz C., Montagnese S., Senzolo M., Mookerjee R. P. (2021). Management of the major complications of cirrhosis: Beyond guidelines. J Hepatol.

[29] United Nations. (2020). Sustainable Development Goals. 2030 Agenda. [Internet].

[30] Xu C. Q., Mohamad Y., Kappus M. R., Boyarsky B., Ganger D. R., Volk M. L. (2021). The relationship between frailty and cirrhosis etiology: From the Functional Assessment in Liver Transplantation (FrAILT) Study. Liver Int.

[31] Buchard B., Boirie Y., Cassagnes L., Lamblin G., Coilly A., Abergel A. (2020). Assessment of Malnutrition, Sarcopenia and Frailty in Patients with Cirrhosis: Which Tools Should We Use in Clinical Practice?. Nutrients.

[32] Tsuchihashi J., Koya S., Hirota K., Koga N., Narao H., Tomita M. (2021). Effects of In-Hospital Exercise on Frailty in Patients with Hepatocellular Carcinoma. Cancers (Basel).

[33] Lin F. P., Visina J. M., Bloomer P. M., Dunn M. A., Josbeno D. A., Zhang X. (2021). Prehabilitation-Driven Changes in Frailty Metrics Predict Mortality in Patients With Advanced Liver Disease. Am J Gastroenterol.

[34] Chen H. W., Ferrando A., White M. G., Dennis R. A., Xie J., Pauly M. (2020). Home-Based Physical Activity and Diet Intervention to Improve Physical Function in Advanced Liver Disease: A Randomized Pilot Trial. Dig Dis Sci.

[35] Lai J. C., Segev D. L., McCulloch C. E., Covinsky K. E., Dodge J. L., Feng S. (2018). Physical frailty after liver transplantation. Am J Transplant.

[36] Lai J. C., Sonnenday C. J., Tapper E. B., Duarte-Rojo A., Dunn M. A., Bernal W. (2019). Frailty in liver transplantation: An expert opinion statement from the American Society of Transplantation Liver and Intestinal Community of Practice. Am J Transplant.

[37] Padhi B. K., Gandhi A. P., Sandeep M., Shamim M. A., De A., Rathi S. (2024). Prevalence of Frailty and Its Impact on Mortality and Hospitalization in Patients With Cirrhosis: A Systematic Review and Meta-analysis. J Clin Exp Hepatol.

[38] Singh S., Taneja S., Tandon P., Bansal A., Gorsi U., Roy A. (2022). A Comparison of Different Frailty Scores and Impact of Frailty on Outcome in Patients With Cirrhosis. J Clin Exp Hepatol.

[39] Raju S., Mathew J. S., Sudhindran S., Padma U. D. (2021). Quality of life 5 years following liver transplantation. Indian J Gastroenterol.

[40] Dunn M. A., Rogal S. S., Duarte-Rojo A., Lai J. C. (2020). Physical Function, Physical Activity, and Quality of Life After Liver Transplantation. Liver Transplant.

[41] Kim N. G., Sharma A., Saab S. (2020). Cardiovascular and metabolic disease in the liver transplant recipient. Best Pract Res Clin Gastroenterol.

[42] Gómez-Bravo M., Prieto Castillo M., Navasa M., Sánchez-Antolín G., Lladó L., Otero A. (2022). Effects of everolimus plus minimized tacrolimus on kidney function in liver transplantation: REDUCE, a prospective, randomized controlled study. Rev Esp Enferm Dig.

[43] Rodríguez-Perálvarez M., Colmenero J., González A., Gastaca M., Curell A., Caballero-Marcos A. (2022). Cumulative exposure to tacrolimus and incidence of cancer after liver transplantation. Am J Transplant.

[44] Millson C., Considine A., Cramp M. E., Holt A., Hubscher S., Hutchinson J. (2020). Adult liver transplantation: UK clinical guideline - part 2: surgery and post-operation. Frontline Gastroenterol.

[45] Deng L. X., Bischoff K. E., Kent D. S., O’Riordan D. L., Pantilat S. Z., Lai J. C. (2021). Frailty is strongly associated with self-reported symptom burden among patients with cirrhosis. Eur J Gastroenterol Hepatol.

[46] Neuberger J. (2022). Long-term Care of the Adult Liver Transplant Recipient. J Clin Exp Hepatol.

[47] McKie P., Webzell I., Tavabie O., Loewenthal D., Heaton N. (2020). An exploratory study of the experiences of deceased-donor liver transplant recipients and their need for psychotherapeutic support. J Clin Nurs.

[48] Fidan C., Akdur A., Kirnap M., H. Selçuk, Yildirim S., Moray G. (2021). Analysis of Quality of Life, Depression, and Sexual Function in Patients on the Liver Transplant List. Turkish J Gastroenterol.

