# Supplementary material for: Liver frailty and impact of liver transplants on transplanted patients’ health
Source: Rev Lat Am Enfermagem. 2025 May 19;33:e4563. doi: 10.1590/1518-8345.7330.4563 (PMC12088654; doi:10.1590/1518-8345.7330.4563)
Supplement: Translation

# Translation: Fragilidade hepática e o impacto do transplante de fígado na saúde dos pacientes com transplante hepático

**DOI:** 10.1590/1518-8345.7330.4564

**Authors:** Victor Fernandez-Alonso, Ana Maria Hernandez-Matias, Manuela Perez-Gomez, Leyre Rodriguez-Leal, Maria Nieves Moro-Tejedor

**Affiliations:** 1 Instituto de Investigación Sanitaria Gregorio Marañón, Madrid, Espanha; 2 Universidad Autónoma de Madrid, Escuela Universitaria de Enfermeria de Cruz Roja, Madrid, Espanha; 3 Hospital General Universitario Gregorio Marañon, Madrid, Espanha

**Keywords:** Fragilidade, Cirrose Hepática, Transplante de Fígado, Alcoólicos, Enfermagem, Reabilitação

## Abstract

analisar o índice de fragilidade hepática em uma coorte de pacientes desde o momento da inclusão na lista de espera até um ano depois do transplante hepático.

estudo de coorte de pacientes incluídos na lista de espera para transplante hepático no período de janeiro de 2020 a dezembro de 2021. Foi realizada análise das variáveis e contraste de hipóteses por meio do teste U de Mann-Whitney e correlação de Spearman, teste de medidas pareadas e análise multivariada.

a amostra foi composta por n=51 pacientes com média de idade de 57,20 anos (DP=9,70), sendo 74,50% homens. O índice médio de fragilidade hepática pré-transplante foi de 3,71 (DP=0,74), sendo mais elevado em pacientes com doença hepática avançada (p=0,004), etiologia relacionada ao álcool (p=0,039) e situação de desemprego (p=0,014). Após o transplante, a fragilidade hepática melhorou (p<0,001), mantendo uma correlação diretamente proporcional com a idade (p=0,014).

doença hepática avançada, etiologia relacionada ao álcool e tempo na lista de espera afetam a fragilidade hepática durante o processo de transplante hepático. Pacientes de mais idade submetidos a transplante hepático são mais frágeis.

## Destaques

**(1)** Pacientes frágeis são aqueles cuja etiologia está relacionada ao álcool. **(2)** Pacientes frágeis são aqueles que estão inativos ou desempregados. **(3)** Após o transplante hepático, a saúde física e mental dos pacientes melhora. **(4)** A etiologia, o MELD-Na e o tempo em lista de espera afetam a fragilidade. **(5)** Os enfermeiros devem liderar o gerenciamento dos fatores de risco cardiovascular.

## Introdução

A cirrose hepática (CH) é o principal diagnóstico para inclusão na lista de espera para transplante hepático (TH) na Espanha^(1)^. A principal causa de cirrose hepática na Europa e na Espanha é a hepatite alcoólica ou doença hepática alcoólica (DHA), seguida pela doença hepática gordurosa não alcoólica (DHGNA) e pelas hepatites virais B e C, cuja prevalência tem diminuído em nosso meio devido aos avanços em sua prevenção e tratamento^(2-3)^.

Em 2022, conforme os dados da Organização Nacional de Transplantes (ONT), foram realizados 1.159 transplantes de fígado na Espanha, o que representa uma taxa de 24,4 por milhão de habitantes. Foram incluídos na lista de espera 1294 pacientes com idade média de 54,6 anos, 54% devido a CH e 38% devido a cirrose por DHA. Na lista de espera, a média de tempo foi de 55 dias, com intervalo interquartil (IIQ) de 15-144 dias, e a mortalidade na lista de espera foi de 2,4%^(1)^. O TH continua sendo a opção de tratamento mais eficaz para todos os tipos de insuficiência hepática, outras etiologias não relacionadas à insuficiência hepática e para o câncer de fígado^(3)^.

Fragilidade é um termo avaliado incialmente em Geriatria, e foi definido como “um estado de maior vulnerabilidade ao estresse físico (por exemplo, cirurgia) e diminuição das reservas fisiológicas”^(4)^. Vários fatores influenciam a fragilidade física, tais como: a gravidade da doença hepática, a idade, a massa muscular, o estado nutricional e as comorbidades não relacionadas ao fígado (diabetes, doença cardíaca, insuficiência renal, etc.)^(4)^. Esta condição inclui sarcopenia (diminuição da massa muscular esquelética), imobilidade progressiva, redução do gasto energético e desnutrição^(5)^. A fragilidade física é uma condição prevalente em pacientes com cirrose e representa uma manifestação clínica de atrofia muscular, desnutrição e comprometimento funcional^(6)^. Existem vários testes para mensurar a fragilidade física em pacientes com doença hepática, dentre os quais é possível destacar o Índice de Fragilidade Hepática (*Liver Frailty Index* - LFI), um teste funcional baseado no desempenho do paciente, considerado de fácil aplicação e de alta precisão^(7-8)^. A fragilidade e a mortalidade de pacientes candidatos a transplante foram recentemente descritas em uma revisão, sendo que a fragilidade hepática apresentou valor preditivo para a mortalidade em lista de espera^(9)^. A prevalência de fragilidade hepática em pacientes candidatos a TH varia entre 15%^(10)^ e 21%^(11)^ e a de pacientes frágeis gira em torno de 60%^(10-11)^. Após o TH, a fragilidade hepática melhora e sua prevalência diminui^(12)^. O estado de fragilidade hepática está estatisticamente relacionado a admissões em unidades de terapia intensiva, maior tempo de internação hospitalar e complicações pós-transplante hepático, além do aumento das despesas econômicas^(11,13)^.

Na prática avançada de Enfermagem Digestiva, os enfermeiros prestam cuidados especializados focados nos sinais e sintomas associados ao estágio cirrótico, com especial atenção à cirrose descompensada^(14)^. Quando o paciente é aceito como candidato ao TH, o enfermeiro realiza uma avaliação integral e desenvolve um plano de cuidados considerando as necessidades de saúde física e mental, a etiologia da doença hepática e os determinantes sociais da saúde. Este plano aborda riscos potenciais durante o período de espera e após o TH.

O principal objetivo deste estudo foi analisar o índice de fragilidade hepática em uma coorte de pacientes, desde o momento da inclusão na lista de espera até um ano depois de realizado o TH, além de determinar o impacto do TH na saúde dos pacientes.

## Método

### Design do estudo

Foi realizado um estudo de coorte longitudinal com *design* observacional, com uma fase retrospectiva e outra prospectiva. Para relatar os resultados, foi utilizado como referência o guia de relatórios *Strengthening the Reporting of Observational Studies in Epidemiology* (STROBE)^(15)^.

### Contexto

O estudo foi realizado na unidade de TH do Hospital Geral Universitário Gregorio Marañón, uma instituição terciária na Comunidade de Madri, Espanha. Este centro hospitalar realiza apenas THs em adultos e a média de THs durante o período de 2018-2021 foi de 42,25^(1)^.

### Amostra/Participantes

A população do estudo consistiu em todos os pacientes adultos incluídos na lista de espera para um primeiro TH eletivo na unidade de TH do Hospital Universitário Geral Gregorio Marañón em Madri, Espanha, de janeiro de 2020 a dezembro de 2021. Os pacientes selecionados foram aceitos pelo comitê de TH como candidatos. Na consulta de avaliação e informação sobre o transplante, eles foram instruídos acerca do estudo e receberam as informações necessárias. Além disso, assinaram o Termo de Consentimento Livre e Esclarecido.

Realizou-se uma amostragem por conveniência. Não foi realizado cálculo amostral pois consideramos que tínhamos capacidade de acessar a totalidade da amostra de pacientes com transplante hepático da unidade onde o estudo foi realizado. Todos os pacientes que receberam esse tipo de transplante nesse período foram incluídos. Pacientes incluídos para TH de urgência foram excluídos por conta das dificuldades para a realização de exames físicos e o preenchimento de questionários devido ao estágio avançado da doença hepática e à presença de encefalopatia hepática grave. Pacientes que já haviam recebido outro transplante de órgão foram excluídos, sendo também retirados do acompanhamento os pacientes que necessitaram de retransplante mais de um mês após o primeiro TH. Finalmente, foram também excluídos os pacientes que não concluíram a coleta de dados durante o período do estudo.

### Variáveis

Foram coletadas variáveis sociodemográficas, a saber: idade, em anos; sexo: masculino/feminino; estado civil: solteiro, em união estável, separado/divorciado ou viúvo; nível de escolaridade: ensino fundamental, médio, superior, profissionalizante ou universitário; situação de emprego: não trabalha-desempregado, ativo ou aposentado-pensionista; e religião: professa alguma religião ou não professa nenhuma religião^(16)^. Também foram coletadas variáveis clínicas associadas aos fatores de risco cardiovascular: obesidade, diabetes *mellitus* (DM), hipertensão arterial, dislipidemia e tabagismo. Variáveis relacionadas à doença hepática foram coletadas no momento da inclusão na lista de espera: prognóstico do paciente com cirrose, índice de fragilidade hepática, nível de ansiedade e depressão e etiologia, determinada por meio de testes analítico-funcionais e sorologias hepáticas. Foram registrados o tempo, em dias, na lista de espera, a admissão hospitalar após TH e a necessidade de readmissão durante o primeiro ano após TH. Doze meses depois do TH as variáveis clínicas e aquelas relacionadas à doença hepática foram reavaliadas.

### Fontes de dados/medidas

A coleta de dados iniciou-se uma vez obtida a aprovação do Comitê de Ética e do centro onde a pesquisa foi realizada e após a obtenção do consentimento assinado pelos participantes. Foi realizada retrospectivamente na primeira visita, acessando-se os registros e o histórico médico dos pacientes incluídos na lista de espera de janeiro de 2020 a fevereiro de 2021. Posteriormente, o enfermeiro de consulta de TH coletou os dados prospectivamente, no momento da inclusão na lista de espera para TH e, 12 meses após o TH, a coleta de dados correspondente foi coordenada com as visitas habituais de acompanhamento dos pacientes transplantados. A equipe de pesquisa foi responsável por coletar dados dos prontuários médicos dos pacientes.

Para completar os questionários e obter o índice de fragilidade hepática, as consultas de acompanhamento clínico foram coordenadas com o protocolo do estudo. Os questionários foram fornecidos pessoalmente pela enfermeira da clínica de TH na chegada do paciente e coletados na saída no mesmo dia. O índice de fragilidade hepática foi calculado na consulta de enfermagem, onde a enfermeira orientou o paciente nos três exames. A enfermeira então completou os dados do índice.

### Índices e instrumentos

#### Liver Frailty Index (LFI)

Todos os pacientes em lista de espera para TH foram submetidos a uma medida objetiva de fragilidade física enquanto estavam na lista, considerando o Índice de Fragilidade Hepática, que é um índice contínuo específico para pacientes com cirrose calculado a partir de pontuações em três testes simples baseados no desempenho:

Força de preensão manual dominante: a média entre três medições usando um dinamômetro de mão, em quilogramas.

Levantar-se da cadeira: número de segundos que um paciente leva para se levantar cinco vezes de uma cadeira com os braços cruzados sobre o peito.

Teste de equilíbrio: número de segundos, com um máximo de 10 segundos, que o paciente consegue permanecer em cada uma das três posições seguintes: lateral, de pé com os pés paralelos; semi-tandem, em pé com um pé ligeiramente à frente do outro; e tandem, em pé com um pé na frente do outro, tocando o calcanhar do pé da frente com o dedo do pé de trás.

Para calcular o índice de fragilidade hepática, foi utilizada a calculadora *online* encontrada em https://liverfrailtyindex.ucsf.edu. A pontuação obtida no índice categoriza o paciente como robusto (<3,2), pré-frágil (3,2-4,4) ou frágil (≥4,5)^(17)^. As medições do índice de fragilidade hepática foram repetidas aos 12 meses, coincidindo com uma visita à consulta ambulatorial pós-transplante. Para este estudo, a avaliação de fragilidade mais próxima da data do transplante foi utilizada como medida de fragilidade pré-transplante.

#### Model for End-Stage Liver Disease-Sodium (MELD-Na)

O Modelo para Doença Hepática Terminal-Sódio (MELD-Na) é um índice prognóstico que avalia a gravidade da cirrose. Calcula-se a partir de uma combinação logarítmica dos valores laboratoriais de bilirrubina, INR, creatinina e sódio sérico. Valores ≥15 indicam maior sobrevida sem TH^(18-19)^.

#### Hospital Anxiety and Depression Scale (HADS)

A Escala Hospitalar de Ansiedade e Depressão (HADS) é um dos instrumentos de autorrelato mais amplamente utilizados para detectar o sofrimento emocional (ansiedade e depressão) em pessoas com doenças físicas^(20)^. É composta por duas subescalas (HAS: Ansiedade e HDS: Depressão) que possuem sete itens classificados em uma escala Likert de 4 pontos (0-3). As pontuações para cada subescala podem variar de 0 a 2. Valores entre 11 e 21 são considerados casos prováveis de ansiedade e/ou depressão^(20)^. Esta escala foi validada em espanhol em uma população hospitalizada, sendo sua consistência interna avaliada através do alfa de Cronbach de 0,90 para a escala completa, 0,84 para a subescala de depressão e 0,85 para a subescala de ansiedade^(21)^. Esta ferramenta tem sido utilizada internacionalmente para avaliação psicológica em pacientes com doença hepática crônica^(22)^ e receptores de transplante hepático^(23)^.

### Vieses

Para evitar vieses, os dados coletados retrospectivamente foram recolhidos pelo mesmo pesquisador e extraídos do prontuário médico digital de cada paciente. Os dados, questionários e testes recolhidos na consulta de Enfermagem também foram coletados pelo mesmo enfermeiro de forma sistemática e rigorosa, evitando assim vieses de observação e interpretação por outros pesquisadores.

### Análise de dados

Os dados foram armazenados em um banco de dados, sendo analisados por meio do *softwareStatistical Package for the Social Sciences* (SPSS), versão 26.0 (SPSS Inc., Chicago, Illinois). Foi realizada uma análise exploratória para identificar valores discrepantes ou extremos e caracterizar diferenças entre grupos de casos. As variáveis sociodemográficas foram recodificadas para facilitar a análise sem perder informações relevantes. A distribuição dos dados foi estudada utilizando o teste de Kolmogorov-Smirnov. Foi realizada uma análise descritiva dos dados e um teste das principais hipóteses empregando o teste U de Mann-Whitney e o teste de correlação de Spearman. As alterações nos índices LFI e MELD-Na foram determinadas com um teste de medidas pareadas. Foi realizada análise multivariada por meio de regressão logística binária para identificar as variáveis que explicam o comportamento da fragilidade hepática antes e depois do transplante. Para facilitar a interpretação, foi construída uma variável dicotômica que classificou os pacientes em dois grupos: frágeis ou pré-frágeis frente aos pacientes robustos. Este modelo incluiu aquelas variáveis que apresentaram relação estatisticamente significativa com a fragilidade na análise univariada. Em todos os casos, apenas valores de p menores que 0,05 foram considerados estatisticamente significativos.

### Considerações éticas

Este estudo, bem como seu consentimento por escrito, foi aprovado pelo Comitê de Ética do Hospital Geral Universitário Gregorio Marañón (código IMPACT_TH do termo 02/2021) e foi realizado de acordo com os princípios articulados na Declaração de Helsinki^(24)^, como assim também no Regulamento (UE) 2016/679 do Parlamento Europeu e do Conselho de 27 de abril de 2016 sobre Proteção de Dados^(25)^, garantindo assim a correta codificação e pareamento dos dados.

## Resultados

A população do estudo foi de aproximadamente n=86. Foram incluídos n=70 pacientes, dos quais n=11 (15,71%) e n=7 (10%) não completaram as avaliações pré e pós-transplante, respectivamente. Além disso, um paciente faleceu após um derrame grave durante o acompanhamento pós-transplante. Portanto, a perda total foi de 19 (27,14%) pacientes. A amostra de pacientes analisada foi n=51 com média de idade de 57,20 anos (DP=9,70), sendo n=38 (74,5%) homens.

### Situação pré-transplante

Em relação às variáveis sociodemográficas, 75,55% (n=37) estavam em união estável, enquanto 13,7% (n=7) eram solteiros, 11,8% (n=6) separados e 2% (n=1) viúvos. 53,6% possuíam um nível académico superior ao ensino obrigatório: 19,6% (n=10) ensino fundamental, 29,4% (n=15) ensino secundário obrigatório, 19,6% (n=10) ensino médio, 21,6% (n=11) formação profissional e 9,8% (n=5) estudos universitários. Dentre os participantes, 35,3% (n=18) não professavam nenhuma religião, enquanto 39,2% (n=20) professavam a religião católica como praticantes, 27,6% (n=9) eram católicos não praticantes e 7,8% (n=4) professavam outras religiões. Finalmente, 29,41% (n=15) tinham emprego, enquanto 35,3% (n=18) estavam desempregados e 35,3% (n=18) estavam aposentados. No momento da inclusão na lista de espera, o LFI apresentou significância estatística com a situação de emprego, sendo menor naqueles pacientes que permaneciam ativos. Em relação às variáveis clínicas e aos fatores de risco cardiovascular no momento da inclusão na lista de espera, 35,29% (n=18) eram diabéticos, 37,25% (n=19) eram hipertensos, 27,45% (n=14) apresentavam dislipidemia, 19,61% (n=10) eram fumantes ativos e 70,59% (n=36) apresentavam sobrepeso ou obesidade (Tabela 1).

**Tabela 1 -:** Análise bivariada das variáveis sociodemográficas e clínicas e do escore do *Liver Frailty Index* pré-transplante. Madri, Espanha, 2020-2021

Variável PreTH* (n ^†^ =51)	Global n ^†^ (%)	LFI ^‡^ x̅ ^§^ (DE ^||^ )	Sig ^¶^ (valor p)
Sexo			
Masculino	38 (74,5%)	3,72 (0,81)	0,897
Feminino	13 (25,5%)	3,67 (0,50)	
Estado civil			
União Estável	37 (72,5%)	3,74 (0,66)	0,533
Sem parceiro	14 (27,5%)	3,62 (0,95)	
Nível de educação			
Ensino médio ou inferior	25 (49%)	3,80 (0,59)	0,371
Ensino superior	26 (51%)	3,62 (0,86)	
Situação de emprego			
Ativo	36 (70,6%)	3,85 (0,69)	**0,014**
Não ativo	15 (29,4%)	3,67 (0,76)	
Religião			
Não professa nenhuma religião	18 (35,3%)	3,80 (0,94)	0,315
Professa alguma religião	33 (64,7%)	3,66 (0,62)	
Diabetes *mellitus*			
Sim	18 (35,3%)	3,95 (0,59)	0,119
Não	33 (64,7%)	3,52 (0,79)	
Hipertensão arterial			
Sim	19 (37,3%)	3,74 (0,78)	0,876
Não	32 (62,7%)	3,69 (0,73)	
Dislipidemia			
Sim	14 (27,5%)	4,02 (0,80)	0,069
Não	37 (72,5%)	3,59 (0,69)	
Tabagismo			
Fuma	10 (19,6%)	3,66 (0,85)	0,822
Não fuma	41 (80,4%)	3,72 (0,85)	
Etiologia			
DHA**	19 (37,3%)	3,98 (0,57)	**0,039**
Não DHA**	32 (62,7%)	3,55 (0,79)	

*PreTH = Pré-transplante hepático; ^†^n = Tamanho da amostra; ^‡^LFI = *Liver Frailty Index*; ^§^x̅ = Média; ^||^DP = Desvio-padrão; ^¶^Sig (valor de p) = Significância estatística U de Mann-Whitney; **DHA = Doença hepática alcoólica

A gravidade da doença hepática segundo o índice MELD-Na obteve pontuação média de 15,04 (DP=6,36), apresentando maior risco em 47,1% (n=24) dos pacientes. O LFI obteve pontuação média de 3,71 (DP=0,74), com os seguintes resultados: 15,7% (n=8) frágeis, 66,7% (n=34) pré-frágeis e 17,6% (n=9) robustos. A etiologia da doença hepática foi relacionada ao consumo de álcool em 37,25% (n=19) dos casos. As indicações de TH mais prevalentes por etiologia foram cirrose DHA com carcinoma hepatocelular (CHC) (15,7% [n=8]) e cirrose DHA (13,7% [n=7]). Entre as indicações de TH por etiologia não DHA, as mais prevalentes foram cirrose por vírus da hepatite C e CHC (17,6% [n=9]), hepatite autoimune (9,8% [n=5]) e somente CHC (5,9% [n=3]).

Em relação à esfera da saúde mental, o escore de ansiedade registrou uma mediana de 6 pontos [IIQ 3-9], sendo 64,7% (n=33) um estado não ansioso, 15,7% (n=8) um estado de risco de ansiedade e 19,6% (n=10) um estado de ansiedade. O escore de depressão teve uma mediana de 4 [IIQ 1-7], com 80,4% (n=41) em estado não depressivo, 13,7% (n=7) em risco de depressão e 5,9% (n=3) em estado depressivo.

O LFI foi diretamente correlacionado com a pontuação do índice MELD-Na. Além disso, destacou que a pontuação da escala HDS foi diretamente correlacionada com a HAS e o índice MELD-Na e inversamente com a idade dos pacientes (Tabela 2).

**Tabela 2 -:** Correlação entre variáveis quantitativas e escore do *Liver Frailty Index* pré-transplante. Madri, Espanha, 2020-2021

TH* **(n** ^†^ **=51** )		**Idade**	**IMC** ^‡^	**MELD-Na** ^§^	**HAS** ^||^	**HDS** ^¶^	LFI**
Idade	r	1	0,058	-0,197	-0,208	**-0,323** ^††^	0,268
	valor p		0,686	0,166	0,142	**0,021**	0,057
IMC ^‡^	r	0,058	1	0,091	-0,123	0,071	0,030
	valor p	0,686		0,524	0,390	0,622	0,835
MELD-Na ^§^	r	-0,197	0,091	1	0,187	**0,315** ^††^	**0,392** ^‡‡^
	valor p	0,166	0,524		0,188	**0,024**	**0,004**
HAS ^||^	r	-0,208	-0,123	0,187	1	**0,570** ^‡‡^	-0,008
	valor p	0,142	0,390	0,188		**<0,001**	0,957
HDS ^¶^	r	**-0,323** ^††^	0,071	**0,315** ^††^	**0,570** ^‡‡^	1	0,098
	valor p	**0,021**	0,622	**0,024**	**<0,001**		0,495
LFI**	r	0,268	0,30	**0,391** ^‡‡^	-0,008	0,098	1
	valor p	0,057	0,835	**0,004**	0,957	0,495	

*TH = Transplante hepático; ^†^n = Tamanho da amostra; ^‡^IMC = Índice de massa corporal; ^§^MELD-Na = *Model for End-Stage Liver Disease-Sodium*; ^||^HAS = *Hospital Anxiety Scale*; ^¶^HDS = *Hospital Depression Scale*; **LFI = *Liver Frailty Index*; ^††^A correlação é significativa no nível de 0,05 (bicaudal); ^‡‡^A correlação é significativa no nível de 0,01 (bicaudal)

### Situação pós-transplante

Os n=51 pacientes foram submetidos a transplante hepático eletivo. Após o TH, n=3 (5,88%) necessitaram de retransplante de emergência devido ao mau funcionamento do enxerto primário. A pontuação média do LFI após TH foi de 3,39 (DP=0,77), com n=2 (3,9%) pacientes frágeis, n=32 (62,7%) pré-frágeis e n=17 (33,3%) robustos (Tabela 3), observando-se uma mudança estatisticamente significativa (p<0,001). Assim, n=15 (29,41%) melhoraram sua fragilidade, n=33 (64,71%) a mantiveram e em n=3 (5,88%) a fragilidade piorou em relação à situação pré-transplante.

A mediana do número de dias na lista de espera para transplante foi de 114 [IIQ 46-202] e a mediana do número de dias correspondentes à admissão hospitalar para o processo de transplante foi de 16 [IIQ 13-22]. Não houve diferença estatisticamente significativa entre a pontuação LFI e o tempo em lista de espera (p=0,124), tampouco com relação ao tempo de internação hospitalar (p=0,630). Embora as diferenças não tenham sido estatisticamente significativas, nossos resultados descrevem que pacientes frágeis tendem a necessitar de internações hospitalares mais longas do que aqueles em estado pré-frágil e/ou robusto.

**Tabela 3 -:** Impacto do transplante hepático antes e depois de um ano. Madri, Espanha, 2020-2021

	PreTH* (%, DP ^‡^ )	PosTH ^†^ (%, DP ^‡^ )	Estatístico	Sig ^§^ (valor p)
Diabetes *mellitus*				
Sim	18 (35,29%)	29 (56,86%)	-3.065	**0,007** ^||^
Não	33 (64,71%)	22 (43,14%)		
Hipertensão arterial				
Sim	19 (37,25%)	33 (64,71%)	-3,253	**0,004** ^||^
Não	32 (62,75%)	18 (35,29%)		
Dislipidemia				
Sim	14 (27,45%)	16 (31,37%)	-0.496	0,804
Não	37 (72,55%)	35 (68,63%)		
Tabagismo ativo				
Sim	10 (19,6%)	5 (9,8%)	1,940	0,058
Não	41 (80,4%)	46 (90,2%)		
LFI ^¶^	3,71 (DP 0,74)	3,39 (DP 0,77)	3,764	<0,001**
MELD-Na ^††^	15,04 (DP 6,36)	9,05 (DP 3,07)	6,041	<0,001**
IMC ^‡‡^	28,07 (DP 4,91)	26,82 (DP 5,57)	2,170	0,035**
HAS ^§§^	6,49 (DP 3,82)	4,94 (DP 3,67)	3,257	0,002**
HDS ^||||^	4,43 (DP 3,59)	3,14 (DP 3,58)	2,520	0,015**

*PreTH = Pré-transplante hepático; ^†^PosTH = Pós-transplante hepático; ^‡^DP = Desvio-padrão; ^§^Sig (p-valor) = Significância estatística; ^||^Teste t de Student; ^¶^LFI = *Liver Frailty Index*; **Qui-quadrado; ^††^MELD-Na = *Model for End-Stage Liver Disease-Sodium*; ^‡‡^IMC = Índice de massa corporal; ^§§^HAS = *Hospital Anxiety Scale*; ^||||^HDS = *Hospital Depression Scale*

Durante o primeiro ano após o TH, 56,9% (n=29) sofreram pelo menos uma internação hospitalar. O LFI foi maior entre aqueles que necessitaram de hospitalização (3,73 [DP=0,72]) em comparação com aqueles que não precisaram de internação (3,68 [DP=0,78]) porque o valor de p foi maior que 0,05. Em relação aos fatores de risco cardiovascular, houve aumento de diabetes e hipertensão arterial. Pelo contrário, foi observada uma melhora estatisticamente significativa no índice de massa corporal, índice de fragilidade hepática e nos escores de ansiedade e depressão (Tabela 3). Além disso, em relação ao escore LFI um ano após o TH, encontramos correlação estatisticamente significativa entre o índice MELD-Na e a idade (Tabela 4), mas não observamos nenhuma relação significativa com as variáveis clínicas. Em relação às diferenças no LFI, a pontuação média pré-transplante foi de 3,71 (DP=0,74) ao passo que a pós-transplante foi de 3,39 (DP=0,77) e, no caso do MELD-Na a pontuação pré-transplante foi de 15,04 (DP=6,36) em comparação com a pós-transplante, que foi de 9,06 (DP=3,0), com diferenças estatisticamente significativas (p<0,001).

**Tabela 4 -:** Correlação entre variáveis quantitativas e pontuação do Índice de Fragilidade Hepática pós-transplante. Madri, Espanha, 2020-2021

TH* **(n** ^†^ **=51)**		**Idade**	**IMC** ^‡^	**MELD-Na** ^§^	**HAS** ^||^	**HDS** ^¶^	LFI**
Idade	r	1	-0,098	0,232	**-0,367** ^††^	-0,221	**0,343** ^‡‡^
	valor p		0,495	0,101	**0,008**	0,119	**0,014**
IMC ^‡^	r	-0,098	1	-0,044	-0,061	0,068	-0,127
	valor p	0,495		0,761	0,672	0,636	0,373
MELD-Na ^§^	r	0,232	-0,044	1	-0,117	-0,260	**0,287** ^‡‡^
	valor p	0,101	0,761		0,414	0,065	**0,041**
TEM ^||^	r	**-0,367** ^††^	-0,061	-0,117	1	**0,528** ^††^	-0,136
	valor p	**0,008**	0,672	0,414		**<0,001**	0,341
HDS ^¶^	r	-0,221	0,068	-0,260	**0,528** ^††^	1	0,146
	valor p	0,119	0,636	0,065	**<0,001**		0,308
LFI**	r	**0,343** ^‡‡^	-0,127	**0,287** ^‡‡^	-0,136	0,146	1
	valor p	**0,014**	0,373	**0,041**	0,341	0,308	

*TH = Transplante hepático; ^†^n = Tamanho da amostra; ^‡^IMC = Índice de massa corporal; ^§^MELD-Na = *Model for End-Stage Liver Disease-Sodium*; ^||^HAS = *Hospital Anxiety Scale*; ^¶^HDS = *Hospital Depression Scale*; **LFI = *Liver Frailty Index*; ^††^A correlação é significativa no nível de 0,01 (bicaudal); ^‡‡^A correlação é significativa no nível de 0,05 (bicaudal)

### Análise multivariada

Na análise de regressão multivariada após o ajuste para covariáveis associadas à fragilidade hepática, o modelo no momento da inclusão na lista de espera para transplante hepático apresentou um pseudo R² de 0,3268, indicando bom ajuste do modelo. Avaliada pela área sob a curva (*Area Under the Curve*, AUC) ROC, a capacidade discriminatória foi de 0,8677, mostrando alta precisão na diferenciação entre os grupos pré-frágil ou frágil vs. robusto. Além disso, o teste de ajuste de Hosmer-Lemeshow não foi significativo [χ²(8)=4,29; [*p* =0,8301], o que confirma que o modelo se ajusta adequadamente aos dados observados. Neste modelo, o índice MELD-Na foi significativamente associado à fragilidade (OR=1,309; IC 95%: 1,05–1,63; *p* =0,017), assim como o tempo na lista de espera para transplante hepático (OR=1,010; IC 95%: 1,00–1,02; *p* =0,042); contudo, a etiologia da doença hepática alcoólica (OR=4,841; IC 95%: 0,48–48,83) não atingiu significância estatística (*p* =0,181). Um ano após o transplante hepático, o modelo apresentou um pseudo R² de 0,1598, refletindo ajuste moderado, com AUC de 0,7630 indicando capacidade discriminativa aceitável. O teste de Hosmer-Lemeshow não foi estatisticamente significativo [χ²(8)=8,12; *p* =0,4220], apoiando assim a validade do modelo; o índice MELD-Na apresentou associação próxima à significância estatística (OR=1,237; IC 95%: 0,97–1,58; *p* =0,091), assim como o tempo em lista de espera (OR=1,006; IC 95%: 1,00–1,01; *p* =0,064), sendo a etiologia relacionada ao álcool a variável que apresentou associação estatisticamente significativa com a fragilidade hepática (OR=4,694; IC 95%: 1,05–20,94; *p* =0,043) (Tabela 5).

**Tabela 5 -:** Variáveis que influenciam a fragilidade hepática no momento da inclusão na lista de espera para transplante e após o primeiro ano pós-transplante hepático. Análise multivariada. Madri, Espanha, 2020-2021

**Variável**	**Inclusão na lista de espera para transplante hepático**					**Um ano após o transplante hepático**				
	**Coeficiente de regressão (β)**	**Erro Padrão**	z*	***Odds Ratio*** **(Intervalo de confiança de 95%)**	**Sig** ^†^ **(valor p)**	**Coeficiente de regressão (β)**	**Erro Padrão**	z*	***Odds Ratio*** **(Intervalo de confiança de 95%)**	**Sig** ^†^ **(valor p)**
MELD-Na ^‡^	0,270	0,113	2.39	1.309 (1.050 – 1.633)	**0,017**	0,213	0,156	1,69	1.237 (0,966 – 1,584)	**0,043**
Tempo na lista de espera (dias)	0,011	0,005	2.03	1.010 (1.000 – 1.021)	**0,042**	0,007	0,003	1,85	1.006 (0,999 – 1,013)	0,064
DHA ^§^	1.577	1.179	1,34	4.841 (0,480 – 48.832)	0,181	1.564	3.581	2.03	4.694 (1.052 – 20.941)	0,091
Constante	-3.634	1.673	-2,17	0,026 (0,001 – 0,701)	0,030	-2.518	0,110	-1,84	0,080 (0,005 – 1,183)	0,066

*z = Estatística de regressão logística; ^†^Sig (valor-p) = Significância estatística; ^‡^MELD-Na = *Model for End-Stage Liver Disease-Sodium;* ^§^DHA = Doença hepática alcoólica

## Discussão

Os pacientes mais frágeis são aqueles com descompensação avançada da função hepática, indicada por uma pontuação MELD-Na alta. Pacientes cuja doença hepática está relacionada ao álcool e que estão desempregados ou inativos são também mais frágeis. Um ano após o TH, a fragilidade segue notadamente relacionada ao índice MELD-Na e à idade, sendo os pacientes com mais idade os mais frágeis.

O LFI sustenta o MELD-Na na previsão da mortalidade em pacientes com disfunção hepática^(26)^. Em nossos resultados, o LFI mantém essa correlação antes e depois do transplante de forma direta e significativa. No momento da inclusão na lista de espera, um LFI mais alto também está significativamente relacionado à DHA. O álcool e a síndrome metabólica pioram a sarcopenia, afetando negativamente os músculos e a sinalização de crescimento e aumentando a resistência à insulina^(27)^. Os enfermeiros hepatologistas devem educar a população sobre as consequências deste problema na doença hepática, aconselhando sobre seus efeitos e prevenindo o consumo e o uso abusivo de álcool^(28)^.

A relação entre a fragilidade hepática, o LFI, o tempo decorrido até o transplante e a cirrose devido ao consumo de álcool em pacientes transplantados é complexa e significativa. Pacientes com cirrose induzida por álcool geralmente apresentam maior grau de fragilidade hepática, o que pode influenciar o tempo de espera para o transplante, piorando potencialmente seu estado geral de saúde e os riscos associados ao TH. A avaliação contínua e o gerenciamento adequado do LFI são essenciais para melhorar os resultados em pacientes transplantados com histórico de doença hepática relacionada ao álcool.

Nossos resultados reforçam a implementação dos Objetivos de Desenvolvimento Sustentável da Agenda 2030, cujo principal objetivo é *garantir vidas saudáveis e promover o bem-estar para todos, em todas as idades* e, em particular, *reforçar a prevenção e o tratamento do abuso de substâncias, e uso nocivo do álcool* ^(29)^. Embora nossos resultados descrevam a etiologia relacionada ao álcool como mais frágil e nossa amostra tenha sido limitada, mantém-se a conclusão do estudo, que propõe que, embora a fragilidade seja mais comum em pacientes com DHA, ela está associada à mortalidade em lista de espera independentemente da etiologia da cirrose e deve ser aplicada a todas as etiologias desta patologia^(30)^.

Pacientes inativos, quer seja por desemprego ou aposentadoria, são mais frágeis. A insuficiência hepática deteriora o estado físico e cognitivo e piora as condições físicas para o desempenho empregatício^(13)^. Sabemos que a fragilidade está relacionada à sarcopenia, à desnutrição e à inatividade; embora possa ser uma consequência, pode também ser um fator agravante^(31)^, portanto é preciso focar a inatividade como um fator de risco para o aumento da própria fragilidade. A literatura descreve a importância dos programas de habilitação ou pré-habilitação em pacientes cirróticos candidatos a transplantes^(32-35)^. Para esses programas, o enfermeiro se posiciona como profissional de referência para avaliar a fragilidade, bem como para a colaboração direta e estreita com os profissionais da Atenção Primária e com a família do paciente e seu ambiente, levando em consideração seu nível socioeconômico e cultural^(7)^. O cuidado de enfermagem especializado em cirrose ou hepatologia está focalizado na nutrição, na educação dos pacientes e cuidadores sobre as possíveis complicações, na conscientização sobre o risco de quedas e nos cuidados com a fragilidade da pele, com monitoramento periódico e revisão da adesão aos medicamentos^(28)^. A Sociedade Americana de Transplante propôs uma avaliação multidimensional da fragilidade na prática diária incluindo quatro medidas: o índice de Karnofsky, as Atividades da Vida Diária (AVD), o Índice de Fragilidade Hepática e a prova de marcha de seis minutos^(36)^. As intervenções para tratar a fragilidade hepática devem focar-se no seguinte: exercício físico, nutrição, tratamento farmacológico e treino cognitivo^(37)^.

Após o TH, pacientes mais frágeis apresentam maior risco de complicações e internações hospitalares mais longas^(11,38)^. Nossos resultados não mostram diferenças significativas entre os grupos de fragilidade, mas sim uma tendência semelhante aos resultados^(11)^. Um ano depois do TH, o LFI está diretamente relacionado apenas à idade, retornando a seu conceito primário de Geriatria. Nossos resultados também mostram que a fragilidade hepática continua sendo impactada pela etiologia da DHA, do MELD-Na e pelo tempo na lista de espera um ano após o TH.

Conforme descrito na literatura científica, a TH melhora a vida e a saúde dos pacientes^(39-40)^. Nossos resultados descrevem uma melhora nas dimensões física e mental, em linha com os resultados de outros estudos. Contudo, devemos estar atentos ao agravamento dos fatores de risco cardiovascular^(39)^. Conforme o tratamento médico centrado em atingir a dose mínima de imunossupressão que permita a viabilidade do enxerto transplantado, prevenindo a síndrome metabólica^(41)^, a disfunção renal^(42)^ e o câncer^(43)^, a partir da Enfermagem, é necessário priorizar o controle e manejo desses sintomas com base em hábitos de saúde modificáveis, dieta, exercícios físicos e prevenção do consumo de substâncias tóxicas como tabaco e álcool, mantendo a adesão ao tratamento imunossupressor^(44)^.

Entre os achados secundários do nosso estudo, destaca-se que a depressão está diretamente associada à ansiedade e ao MELD-Na durante o período de espera na lista, ressaltando que aqueles pacientes com doença hepática mais avançada e saúde física precária também sofrem o comprometimento de sua saúde mental. Esses resultados já foram descritos, concluindo-se a necessidade de avaliar a depressão em pacientes frágeis^(37,45)^. Nossos resultados se correlacionam indiretamente com a idade, descrevendo que, no momento da inclusão na lista de espera, pacientes mais jovens apresentam maiores escores de depressão. Um ano após o TH é a pontuação de ansiedade que se correlaciona indiretamente com a idade. Nossos resultados apresentam que a esfera da saúde mental em pacientes mais jovens é mais afetada. Este impacto pode ser devido aos medos e complicações de ter que se submeter a TH quando jovem, às consequências na sua vida pessoal e profissional, ao impacto social e à possível recorrência da doença hepática^(46-48)^.

Os enfermeiros devem concentrar suas intervenções na prevenção do alcoolismo e na educação da população sobre suas consequências para a saúde. Eles também devem liderar programas de reabilitação física ou pré-habilitação, promovendo exercícios físicos direcionados em pacientes frágeis para melhorar sua qualidade de vida e prevenir complicações após o transplante. A saúde mental dos pacientes deve ser avaliada durante todo o processo, com foco nos pacientes mais jovens. O cuidado de enfermagem ao paciente transplantado deve ser direcionado ao manejo dos fatores de risco cardiovascular por meio da mudança de hábitos como alimentação, prática de exercícios físicos e prevenção do consumo de substâncias tóxicas como tabaco e álcool.

Como ponto forte do nosso estudo, podemos citar a realização de acompanhamento prospectivo utilizando índices e instrumentos recentemente implantados em unidades de TH. As principais limitações do estudo seriam o tamanho da amostra, o fato de ter sido desenvolvido em um único centro, as perdas durante o processo de coleta de dados e a rejeição de pacientes incluídos como transplantes de urgência. Além disso, não foram coletados dados sociodemográficos um ano após o TH, o que limitou a análise dos resultados.

## Conclusão

O índice de fragilidade hepática descreve o impacto físico da doença hepática e melhora após o TH. Pacientes com doença hepática avançada, etiologia relacionada ao álcool e situação de desemprego são mais frágeis durante o período pré-transplante. Após o TH, os fatores de risco cardiovascular, diabetes e hipertensão arterial pioram. A saúde mental e a fragilidade dos pacientes melhoram após o TH, sendo mais frágeis os pacientes de idade mais avançada. A etiologia relacionada ao álcool, o MELD-Na e o tempo em lista de espera afetam a fragilidade durante todo o processo de TH.

# Translation: Fragilidad hepática e impacto del trasplante hepático en la salud de pacientes trasplantados hepáticos

**DOI:** 10.1590/1518-8345.7330.4562

**Authors:** Victor Fernandez-Alonso, Ana Maria Hernandez-Matias, Manuela Perez-Gomez, Leyre Rodriguez-Leal, Maria Nieves Moro-Tejedor

**Affiliations:** 1 Instituto de Investigación Sanitaria Gregorio Marañón, Madrid, España; 2 Universidad Autónoma de Madrid, Escuela Universitaria de Enfermeria de Cruz Roja, Madrid, España; 3 Hospital General Universitario Gregorio Marañon, Madrid, España

**Keywords:** Fragilidad, Cirrosis Hepática, Trasplante de Hígado, Alcohólicos, Enfermería, Rehabilitación

## Abstract

analizar el índice de fragilidad hepática en una cohorte de pacientes desde su inclusión en la lista de espera hasta un año después del trasplante hepático.

estudio de cohorte de pacientes incluidos en lista de espera para trasplante hepático desde enero de 2020 hasta diciembre de 2021. Se realizó un análisis de las variables y un contraste de hipótesis mediante las pruebas U de Mann-Whitney y correlación de Spearman, test de medidas pareadas y análisis multivariante.

la muestra fue de n=51 pacientes con una media de edad de 57,20 años (DE=9,70), siendo 74,50% hombres. El índice de fragilidad hepática medio pretrasplante fue 3,71 (DE=0,74), siendo más elevado en pacientes con enfermedad hepática avanzada (p=0,004), etiología relacionada con el alcohol (p=0,039) y situación de desempleo (p=0,014). Tras el trasplante, la fragilidad hepática mejoró (p<0,001), manteniendo una correlación directamente proporcional con la edad (p=0,014).

enfermedad hepática avanzada, etiología relacionada con el alcohol y tiempo en lista de espera impactan en la fragilidad hepática durante el proceso del trasplante hepático. Los pacientes trasplantados hepáticos de más edad son más frágiles.

## Destacados

**(1)** Los pacientes frágiles son aquellos cuya etiología está relacionada con el alcohol. **(2)** Los pacientes frágiles son aquellos que están inactivos o en situación de desempleo. **(3)** Tras el trasplante hepático, la salud física y mental de los pacientes mejora. **(4)** La etiología, el MELD-Na y el tiempo en lista de espera impactan en la fragilidad. **(5)** Las enfermeras deben dirigir el manejo de factores de riesgo cardiovascular.

## Introducción

La cirrosis hepática (CH) es el principal diagnóstico de inclusión en lista de espera para trasplante hepático (TH) en España^(1)^. La principal causa de cirrosis hepática en Europa y España es la hepatitis alcohólica o enfermedad hepática relacionada con el alcohol (EHRA), seguida de la hepatitis grasa no alcohólica (HGNA) y las hepatitis virales B y C, cuya prevalencia ha disminuido en nuestro medio debido a los avances en su prevención y tratamiento^(2-3)^.

En 2022, según los datos de la Organización Nacional de Trasplantes (ONT), en España se realizaron 1159 TH, lo que representa una tasa de 24,4 por millón de habitantes. Se incluyeron en lista de espera 1294 pacientes con una media de edad de 54,6 años, siendo el 54% por CH y el 38% debido a cirrosis por EHRA. En lista de espera, la mediana de tiempo fue de 55 días, con rango intercuartílico (RIC) de 15-144 días, y la mortalidad en lista de espera fue del 2,4%^(1)^. El TH continúa siendo el tratamiento de elección más efectivo para todo tipo de insuficiencia hepática, otras etiologías no relacionadas con insuficiencia hepática y cáncer de hígado^(3)^.

Fragilidad es un término inicialmente evaluado desde la Geriatría, y fue definida como “un estado de mayor vulnerabilidad al estrés físico (por ejemplo, cirugía) y descenso de las reservas fisiológicas”^(4)^. Diversos factores influyen en la fragilidad física, tales como gravedad de la enfermedad hepática, edad, masa muscular, estado nutricional y comorbilidades no relacionadas con el hígado (diabetes, enfermedades cardíacas, insuficiencia renal, etc.)^(4)^. Esta condición abarca sarcopenia (disminución de la masa del músculo esquelético), inmovilidad progresiva, reducción del gasto energético y desnutrición^(5)^. La fragilidad física es una condición prevalente en pacientes con cirrosis y representa una manifestación clínica de atrofia muscular, desnutrición y deterioro funcional^(6)^. Existen varias pruebas y/o *tests* para medir la fragilidad física en pacientes con enfermedad hepática, entre las cuales se destaca el Índice de Fragilidad Hepática (*Liver Frailty Index*, LFI), una prueba funcional basada en el rendimiento del paciente que se considera de fácil aplicación y alta precisión^(7-8)^. La fragilidad y la mortalidad de los pacientes candidatos a trasplante han sido descritas recientemente en una revisión, resultando la fragilidad hepática un valor predictivo de mortalidad en lista de espera^(9)^. La prevalencia de fragilidad hepática en pacientes candidatos a TH oscila entre el 15%^(10)^ y el 21%^(11)^ y la de pacientes frágiles es de en torno al 60%^(10-11)^. Después del TH, la fragilidad hepática mejora y su prevalencia desciende^(12)^. El estado de fragilidad hepática se relaciona estadísticamente con ingresos en unidades de cuidados intensivos, estancias hospitalarias más prolongadas y complicaciones postrasplante hepático, además de un aumento de los costes económicos^(11,13)^.

En la práctica avanzada de Enfermería Digestiva, las enfermeras ofrecen cuidados especializados centrados en los signos y síntomas asociados al estadio cirrótico, prestando especial atención a la cirrosis descompensada^(14)^. Cuando el paciente es aceptado como candidato para TH, la enfermera lleva a cabo una evaluación integral y desarrolla un plan de cuidados en el que se consideran las necesidades físicas y de salud mental, la etiología de la enfermedad hepática y los determinantes sociales de la salud. En este plan se abordan los posibles riesgos tanto durante el periodo de espera como después del TH.

El objetivo principal de este estudio fue analizar el índice de fragilidad hepática en una cohorte de pacientes, desde el momento de su inclusión en la lista de espera hasta un año después del TH, además de determinar el impacto del TH en la salud de los pacientes.

## Método

### Diseño del estudio

Se llevó a cabo un estudio de cohorte longitudinal y diseño observacional, con una fase retrospectiva y otra prospectiva. Para el reporte de los resultados se utilizó la guía de reporte de resultados *Strengthening the Reporting of Observational studies in Epidemiology*(STROBE)^(15)^ como referencia.

### Contexto

El estudio se llevó cabo en la unidad de TH del Hospital General Universitario Gregorio Marañón, una institución de tercer nivel de la Comunidad de Madrid, España. Este centro hospitalario realiza únicamente TH en adultos y la media de TH durante el periodo 2018-2021 fue de 42,25^(1)^.

### Muestra/Participantes

La población de estudio estuvo compuesta por todos los pacientes adultos incluidos en lista de espera para un primer TH electivo en la unidad de TH del Hospital General Universitario Gregorio Marañón de Madrid, España, desde enero de 2020 hasta diciembre de 2021. Los pacientes que fueron seleccionados una vez fueron aceptados por el comité de TH como candidatos. En la consulta de evaluación e información sobre el trasplante se les informó sobre el estudio y se les facilitó la información; además, firmaron el consentimiento informado.

La muestra del estudio fue por conveniencia. No se realizó ningún cálculo muestral porque consideramos que teníamos la capacidad de acceder a toda la muestra de pacientes trasplantados hepáticos de la unidad donde se realizó el estudio. Se incluyó a todos los pacientes que recibieron estos tipos de trasplante durante dicho periodo. También se excluyó a los pacientes que fueron incluidos para TH urgente debido a las dificultades para realizar las pruebas físicas y cumplimentación de cuestionarios por el estado avanzado de enfermedad hepática y la presencia de encefalopatía hepática grave. Se excluyeron pacientes que ya habían recibido otro trasplante de órganos y se retiró del seguimiento a aquellos pacientes que requirieron un retrasplante con una diferencia mayor de 1 mes desde el primer TH. Finalmente, se excluyó a aquellos pacientes que no cumplieron la recogida de datos completa durante el período del estudio.

### Variables

Se recogieron variables sociodemográficas, a saber: edad, en años; sexo: **masculino/femenino; estado civil: soltero, en pareja-casado, separado-divorciado o viudo; nivel de estudios: primaria, secundaria, bachillerato, formación profesional o universitaria; situación de empleo: no trabaja-desempleado, activo o jubilado-pensionista; y religión: profesa alguna religión o no profesa ninguna religión^(16)^. También se recolectaron variables clínicas asociadas a factores de riesgo cardiovascular: obesidad, diabetes *mellitus*** (DM), hipertensión arterial, dislipidemia y tabaquismo. Se recogieron variables relacionadas con la enfermedad hepática al momento de inclusión en la lista de espera: pronóstico del paciente con cirrosis, índice de fragilidad hepática, nivel de ansiedad y depresión y etiología, determinada a través de pruebas analítico-funcionales y serologías hepáticas. Se recogieron los tiempos, en días, en lista de espera, ingreso hospitalario tras el TH y necesidad de reingreso durante el primer año posterior al TH. Tras 12 meses del TH, se volvieron a evaluar las variables clínicas y aquellas relacionadas con la enfermedad hepática.

### Fuentes de datos/Medidas

La recogida de datos se inició al obtener la aprobación del Comité de Ética y del centro donde se realizó la investigación, y habiendo obtenido el consentimiento firmado de los participantes. Se realizó en primera estancia retrospectivamente accediendo a los registros y a la historia clínica de los pacientes incluidos en lista de espera desde enero de 2020 hasta febrero de 2021. Posteriormente, la enfermera de la consulta de TH recogió los datos de manera prospectiva en dicha ocasión al momento de inclusión en lista de espera para TH y, a los 12 meses del TH, se coordinó la recogida de datos correspondiente con las visitas de seguimiento habitual de los pacientes trasplantados. El equipo de investigación se encargó de recoger los datos de las historias clínicas de los pacientes.

Para la cumplimentación de los cuestionarios y la realización del índice de fragilidad hepática, se coordinaron las visitas de seguimiento clínico con el protocolo del estudio. Los cuestionarios fueron facilitados por la enfermera de la consulta de TH mano a mano a la llegada del paciente y recogidos a la salida en el mismo día. El índice de fragilidad hepática se realizó en la consulta de Enfermería, en la que la enfermera guio al paciente en las tres pruebas. A continuación, la enfermera completó los datos del índice.

### Índices e instrumentos

#### Liver Frailty Index (LFI)

Todos los pacientes en lista de espera para TH se sometieron a una medida objetiva de fragilidad física mientras estaban en dicha lista utilizando el índice de fragilidad hepática, que es un índice continuo específico para pacientes con cirrosis calculado a partir de las puntuaciones de tres pruebas simples basadas en el rendimiento:

Fuerza de prensión manual dominante: el promedio de tres mediciones utilizando un dinamómetro de mano, en kilogramos.

Ponerse de pie sobre una silla: la cantidad de segundos que le toma a un paciente levantarse cinco veces en una silla con los brazos cruzados sobre el pecho.

Prueba de equilibrio: la cantidad de segundos, durante un máximo de 10 segundos, que el paciente puede mantenerse en cada una de las siguientes tres posiciones: lateral, de pie con los pies en paralelo; semi-tándem, de pie con un pie ligeramente adelantado al otro; y tándem, de pie con un pie delante del otro tacando el talón del pie de adelante con la puntera del pie de atrás.

Para calcular el índice de fragilidad hepática, se utilizó la calculadora en línea que se encuentra en http://liverfrailtyindex.ucsf.edu. La puntación obtenida en el índice categoriza al paciente como robusto (<3,2), pre-frágil (3,2-4,4) o frágil (≥4,5)^(17)^. Las mediciones del índice de fragilidad hepática se repitieron a los 12 meses, coincidiendo con una visita a la consulta ambulatoria postrasplante. Para este estudio, se utilizó la evaluación de fragilidad más cercana a la fecha del trasplante como medida de fragilidad pretrasplante.

#### Model for End-Stage Liver Disease-Sodium (MELD-Na)

El Modelo de Enfermedad Hepática Terminal-Sodio (MELD-Na) es un índice pronóstico que evalúa la gravedad de la cirrosis. Su cálculo es una combinación logarítmica de valores de laboratorio de bilirrubina, INR, creatinina y sodio sérico. Valores ≥15 indican mayor supervivencia sin TH^(18-19)^.

#### Hospital Anxiety and Depression Scale (HADS)

La Escala Hospitalaria de Ansiedad y Depresión (HADS) es uno de los instrumentos de autoinforme más utilizados para detectar malestar emocional (ansiedad y depresión) en personas con enfermedades físicas^(20)^. Consta de dos subescalas (HAS: Ansiedad y HDS: Depresión) que tienen siete ítems calificados mediante una escala Likert de 4 puntos (0-3). Las puntuaciones para cada subescala pueden variar de 0 a 2. Los valores entre 11 y 21 se consideran probables casos de ansiedad y/o depresión^(20)^. Esta escala fue validada al español en población hospitalizada, siendo su consistencia interna evaluada mediante alfa de Cronbach de 0,90 para la escala completa, 0,84 para la subescala de depresión y 0,85 para la subescala de ansiedad^(21)^. Esta herramienta se ha empleado internacionalmente para la evaluación psicológica en pacientes con enfermedad hepática crónica^(22)^ y trasplantados hepáticos^(23)^.

### Sesgos

Para evitar sesgos, los datos recogidos de manera retrospectiva fueron recolectados por el mismo investigador y extraídos de la historia clínica digital de cada paciente. Los datos, cuestionarios y pruebas recogidos en la consulta de Enfermería también fueron recogidos por la misma enfermera de manera sistematizada y rigurosa, evitando así sesgos de observación e interpretación de otros investigadores.

### Análisis de datos

 Los datos se almacenaron en una base de datos y se analizaron utilizando el *software* *Statistical Package for the Social Sciences* (SPSS), versión 26.0 del paquete estadístico (SPSS Inc., Chicago, Illinois). Se realizó un análisis exploratorio para identificar valores atípicos o extremos y caracterizar las diferencias entre grupos de casos. Se recodificaron las variables sociodemográficas para facilitar el análisis sin perder información relevante. Se estudió la distribución de los datos con la prueba de Kolmogorov-Smirnov. Se realizó un análisis descriptivo de los datos y un contraste de las hipótesis principales utilizando las pruebas U de Mann-Whitney y de correlación de Spearman. Los cambios en los índices LFI y MELD-Na se determinaron con un *test* de medidas pareadas. Se realizó un análisis multivariante mediante regresión logística binaria para identificar las variables que explicaron el comportamiento de la fragilidad hepática antes y después del trasplante. Para facilitar la interpretación, se construyó una variable dicotómica que clasificaba a los pacientes en dos grupos: frágiles o pre-frágiles frente a robustos. En este modelo se incluyeron aquellas variables que mostraron una relación estadísticamente significativa con la fragilidad en el análisis univariante. En todos los casos, solo se consideraron estadísticamente significativos valores p inferiores a 0,05. 

### Consideraciones éticas

Este estudio, al igual que su consentimiento escrito, fue aprobado por el Comité de Ética del Hospital General Universitario Gregorio Marañón (código IMPACT_TH del acta 02/2021) y se llevó a cabo de acuerdo con los principios articulados en la Declaración de Helsinki^(24)^, así como en el Reglamento (UE) 2016/679 del Parlamento Europeo y los del Consejo de 27 de abril de 2016 de Protección de Datos^(25)^, asegurando de esta manera una correcta codificación y emparejamiento de datos.

## Resultados

La población de estudio fue aproximadamente de n=86. Se incluyó a un total de n=70 pacientes, de los cuales n=11 (15,71%) y n=7 (10%) no cumplimentaron las evaluaciones pretrasplante y postrasplante, respectivamente. Además, un paciente falleció tras un accidente cerebrovascular masivo durante el seguimiento postrasplante. Por lo tanto, el total de pérdidas fue de 19 (27,14%) pacientes. La muestra de pacientes analizada fue de n=51 con una media de edad de 57,20 años (DE=9,70), siendo n=38 (74,5%) hombres.

### Situación pretrasplante

Respecto a las variables sociodemográficas, el 75,55% (n=37) estaba en pareja mientras que el 13,7% (n=7) era soltero, el 11,8% (n=6) estaba separado y el 2% (n=1) viudo/a. El 53,6% tenía un nivel académico superior a la educación obligatoria: 19,6% (n=10) educación primaria, 29,4% (n=15) educación secundaria, 19,6% (n=10) bachillerato, 21,6% (n=11) formación profesional y 9,8% (n=5) estudios universitarios. El 35,3% (n=18) no profesaba ninguna religión, mientras que el 39,2% (n=20) profesaba la religión católica como practicante, el 27,6% (n=9) era católico no practicante y el 7,8% (n=4) profesaba otras religiones. Por último, el 29,41% (n=15) tenía trabajo, mientras que el 35,3% (n=18) estaba desempleado y el 35,3% (n=18) estaba jubilado. Al momento de inclusión en lista de espera, el LFI muestra significancia estadística con la situación laboral, siendo menor en aquellos pacientes que se mantienen activos. En cuanto a las variables clínicas y los factores de riesgo cardiovascular al momento de inclusión en lista de espera, el 35,29% (n=18) era diabético, el 37,25% (n=19) era hipertenso, el 27,45% (n=14) tenía dislipidemia, el 19,61% (n=10) era fumador activo y el 70,59% (n=36) tenía sobrepeso u obesidad (Tabla 1).

**Tabla 1 -:** Análisis bivariante de las variables sociodemográficas y clínicas y la puntuación del *Liver Frailty Index* pretrasplante. Madrid, España, 2020-2021

Variable PreTH* (n ^†^ =51)	Global n ^†^ (%)	LFI ^‡^ x̅ ^§^ (DE *||* )	Sig ^¶^ (valor p)
Sexo			
Masculino	38 (74,5%)	3,72 (0,81)	0,897
Femenino	13 (25,5%)	3,67 (0,50)	
Estado civil			
En pareja	37 (72,5%)	3,74 (0,66)	0,533
Sin pareja	14 (27,5%)	3,62 (0,95)	
Nivel de estudios			
Secundario o inferior	25 (49%)	3,80 (0,59)	0,371
Educación superior	26 (51%)	3,62 (0,86)	
Situación de empleo			
Activo	36 (70,6%)	3,85 (0,69)	**0,014**
No activo	15 (29,4%)	3,67 (0,76)	
Religión			
No profesa ninguna religión	18 (35,3%)	3,80 (0,94)	0,315
Profesa alguna religión	33 (64,7%)	3,66 (0,62)	
Diabetes *mellitus*			
Sí	18 (35,3%)	3,95 (0,59)	0,119
No	33 (64,7%)	3,52 (0,79)	
Hipertensión arterial			
Sí	19 (37,3%)	3,74 (0,78)	0,876
No	32 (62,7%)	3,69 (0,73)	
Dislipidemia			
Sí	14 (27,5%)	4,02 (0,80)	0,069
No	37 (72,5%)	3,59 (0,69)	
Hábito tabáquico			
Fuma	10 (19,6%)	3,66 (0,85)	0,822
No fuma	41 (80,4%)	3,72 (0,85)	
Etiología			
EHRA**	19 (37,3%)	3,98 (0,57)	**0,039**
No EHRA**	32 (62,7%)	3,55 (0,79)	

*PreTH = Pretrasplante hepático; ^†^n = Tamaño de la muestra; ^‡^LFI = *Liver Frailty Index*; ^§^x̅*=*Media; ^||^DE = Desviación estándar; ^¶^Sig (valor p) = Significancia estadística U de Mann-Whitney; **EHRA = Enfermedad hepática relacionada con el alcohol

La gravedad de la enfermedad hepática según el índice MELD-Na obtuvo una puntación media de 15,04 (DE=6,36), presentando mayor riesgo en el 47,1% (n=24) de los pacientes. El LFI obtuvo una puntuación media de 3,71 (DE=0,74), con los siguientes resultados: 15,7% (n=8) frágiles, 66,7% (n=34) pre-frágiles y 17,6% (n=9) robustos. La etiología de la enfermedad hepática estuvo relacionada con el consumo de alcohol en el 37,25% (n=19) de los casos. Las indicaciones de TH por etiología más prevalentes fueron cirrosis EHRA con carcinoma hepatocelular (CHC) (15,7% [n=8]) y cirrosis EHRA (13,7% [n=7]). De entre las indicaciones de TH por etiología no EHRA, las más prevalentes fueron cirrosis por virus de la hepatitis C y CHC (17,6% [n=9]), hepatitis autoinmune (9,8% [n=5]) y únicamente CHC (5,9% [n=3]).

En cuanto a la esfera de la salud mental, la puntación de ansiedad registró una mediana de 6 puntos [RIC 3-9], siendo 64,7% (n=33) estado no ansioso, 15,7% (n=8) estado de riesgo de ansiedad y 19,6% (n=10) estado de ansiedad. La puntuación de depresión obtuvo una mediana de 4 [RIC 1-7], siendo 80,4% (n=41) estado no depresivo, 13,7% (n=7) riesgo de depresión y 5,9% (n=3) estado de depresión.

El LFI se correlacionó directamente con la puntuación del índice MELD-Na. Además, destacó que la puntuación de la escala HDS se correlacionó directamente con la HAS y el índice MELD-Na e inversamente con la edad de los pacientes (Tabla 2).

**Tabla 2 -:** Correlación entre las variables cuantitativas y la puntación del *Liver Frailty Index* pretrasplante. Madrid, España, 2020-2021

TH* (n ^†^ **=51** )		**Edad**	**IMC** ^‡^	**MELD-Na** ^§^	**HAS** ^||^	**HDS** ^¶^	LFI**
Edad	r	1	0,058	-0,197	-0,208	**-0,323** ^††^	0,268
	valor p		0,686	0,166	0,142	**0,021**	0,057
IMC ^‡^	r	0,058	1	0,091	-0,123	0,071	0,030
	valor p	0,686		0,524	0,390	0,622	0,835
MELD-Na ^§^	r	-0,197	0,091	1	0,187	**0,315** ^††^	**0,392** ^‡‡^
	valor p	0,166	0,524		0,188	**0,024**	**0,004**
HAS ^||^	r	-0,208	-0,123	0,187	1	**0,570** ^‡‡^	-0,008
	valor p	0,142	0,390	0,188		**<0,001**	0,957
HDS ^¶^	r	**-0,323** ^††^	0,071	**0,315** ^††^	**0,570** ^‡‡^	1	0,098
	valor p	**0,021**	0,622	**0,024**	**<0,001**		0,495
LFI**	r	0,268	0,30	**0,391** ^‡‡^	-0,008	0,098	1
	valor p	0,057	0,835	**0,004**	0,957	0,495	

*TH = Trasplante hepático; ^†^n = Tamaño de la muestra; ^‡^IMC = Índice de masa corporal; ^§^MELD-Na = *Model for End-Stage Liver Disease-Sodium*; ^||^HAS = *Hospital Anxiety Scale*; ^¶^HDS*= Hospital Depression Scale*; **LFI = *Liver Frailty Index*; ^††^La correlación es significativa al nivel de 0,05* *(bilateral); ^‡‡^La* *correlación es significativa al nivel de* *0,01* *(bilateral)

### Situación postrasplante

Los n=51 pacientes fueron trasplantados hepáticos electivos. Tras el TH, n=3 (5,88%) precisaron un retrasplante de urgencia por malfunción primaria del injerto. La puntuación LFI media del después del TH fue de 3,39 (DE=0,77), siendo n=2 (3,9%) frágiles, n=32 (62,7%) pre-frágiles y n=17 (33,3%) robustos (Tabla 3), observándose un cambio estadísticamente significativo (p<0,001). Así, n=15 (29,41%) mejoran la fragilidad, n=33 (64,71%) la mantienen y, en n=3 (5,88%), la fragilidad empeora frente a la situación pretrasplante.

**Tabla 3 -:** Impacto del trasplante hepático antes y al año. Madrid, España, 2020-2021

	PreTH*(%, DE ^‡^ )	PosTH ^†^ (%, DE ^‡^ )	Estadístico	Sig ^§^ (valor p)
Diabetes *mellitus*				
Sí	18 (35,29%)	29 (56,86%)	-3.065	**0,007** ^||^
No	33 (64,71%)	22 (43,14%)		
Hipertensión arterial				
Sí	19 (37,25%)	33 (64,71%)	-3,253	**0,004** ^||^
No	32 (62,75%)	18 (35,29%)		
Dislipidemia				
Sí	14 (27,45%)	16 (31,37%)	-0.496	0,804
No	37 (72,55%)	35 (68,63%)		
Tabaquismo activo				
Sí	10 (19,6%)	5 (9,8%)	1,940	0,058
No	41 (80,4%)	46 (90,2%)		
LFI ^¶^	3,71 (DE 0,74)	3,39 (DE 0,77)	3,764	<0,001**
MELD-Na ^††^	15,04 (DE 6,36)	9,05 (DE 3,07)	6,041	<0,001**
IMC ^‡‡^	28,07 (DE 4,91)	26,82 (DE 5,57)	2,170	0,035**
HAS ^§§^	6,49 (DE 3,82)	4,94 (DE 3,67)	3,257	0,002**
HDS ^||||^	4,43 (DE 3,59)	3,14 (DE 3,58)	2,520	0,015**

*PreTH = Pretrasplante hepático; ^†^PosTH = Postrasplante hepático; ^‡^DE = Desviación estándar; ^§^Sig (valor p) = Significancia estadística; ^||^t de Student; ^¶^LFI = *Liver Frailty Index*; **Chi cuadrado; ^††^MELD-Na = *Model for End-Stage Liver Disease-Sodium*; ^‡‡^IMC =*Índice de masa corporal*; ^§§^HAS = *Hospital Anxiety Scale*; ^||||^HDS = *Hospital Depression Scale*

La mediana de días en lista de espera para trasplante fue de 114 [RIC 46-202] y la mediana de días correspondiente al ingreso hospitalario del proceso del trasplante fue de 16 [RIC 13-22]. No se registró ninguna diferencia estadísticamente significativa entre la puntuación LFI y el tiempo en lista de espera (p=0,124). Tampoco en cuanto al tiempo de ingreso hospitalario (p=0,630). Aunque las diferencias no fueron estadísticamente significativas, nuestros resultados describen que los pacientes frágiles tienen tendencia a necesitar ingresos hospitalarios más prolongados que aquellos en los estados pre-frágil y robusto.

Durante el primer año tras el TH, el 56,9% (n=29) sufrió al menos un ingreso hospitalario. El LFI fue mayor entre aquellos que debieron ser hospitalizados (3,73 [DE=0,72]) frente a los que no necesitaron hospitalización (3,68 [DE=0,78]) porque el valor p fue superior a 0,05. Con relación a los factores de riesgo cardiovascular, se produjo un aumento de diabetes e hipertensión arterial. En contraposición, se observó una mejoría estadísticamente significativa respecto al índice de masa corporal, al índice de fragilidad hepática y a las puntuaciones de ansiedad y depresión (Tabla 3). Además, respecto a la puntuación LFI al año del TH, encontramos una correlación estadísticamente significativa con el índice MELD-Na y la edad (Tabla 4), pero no observamos ninguna relación significativa con las variables clínicas. En cuanto a las diferencias en el LFI, la puntuación pretrasplante media fue de 3,71 (DE=0,74) vs. postrasplante, que fue 3,39 (DE=0,77) y, en el caso de MELD-Na pretrasplante (15,04 [DE=6,36]) vs. postrasplante, que fue 9.06 (DE=3,0), siendo las diferencias estadísticamente significativas (p<0,001).

**Tabla 4 -:** Correlación entre las variables cuantitativas y la puntación del *Liver Frailty Index* postrasplante. Madrid, España, 2020-2021

TH* (n ^†^ **=51)**		**Edad**	**IMC** ^‡^	**MELD-Na** ^§^	**HAS** ^||^	**HDS** ^¶^	LFI**
Edad	r	1	-0,098	0,232	**-0,367** ^††^	-0,221	**0,343** ^‡‡^
	valor p		0,495	0,101	**0,008**	0,119	**0,014**
IMC ^‡^	r	-0,098	1	-0,044	-0,061	0,068	-0,127
	valor p	0,495		0,761	0,672	0,636	0,373
MELD-Na ^§^	r	0,232	-0,044	1	-0,117	-0,260	**0,287** ^‡‡^
	valor p	0,101	0,761		0,414	0,065	**0,041**
HAS ^||^	r	**-0,367** ^††^	-0,061	-0,117	1	**0,528** ^††^	-0,136
	valor p	**0,008**	0,672	0,414		**<0,001**	0,341
HDS ^¶^	r	-0,221	0,068	-0,260	**0,528** ^††^	1	0,146
	valor p	0,119	0,636	0,065	**<0,001**		0,308
LFI**	r	**0,343** ^‡‡^	-0,127	**0,287** ^‡‡^	-0,136	0,146	1
	valor p	**0,014**	0,373	**0,041**	0,341	0,308	

*TH = Trasplante hepático; ^†^n = Tamaño de la muestra; ^‡^IMC = Índice de masa corporal; ^§^MELD-Na = *Model for End-Stage Liver Disease-Sodium*; ^||^HAS = *Hospital Anxiety Scale*; ^¶^HDS = *Hospital Depression Scale*; **LFI = *Liver Frailty Index*; ^††^La* *correlación es significativa al nivel de 0,01 (bilateral); ^‡‡^La correlación es significativa al nivel de 0,05 (bilateral)

### Análisis multivariante

En el análisis multivariante de regresión después del ajuste de las covariables asociadas con la fragilidad hepática, el modelo al momento de inclusión en la lista de espera para trasplante hepático presentó un pseudo R² de 0.3268, lo que indica buen ajuste del modelo. Evaluada mediante el área bajo la curva (*Area Under the Curve*, AUC) ROC, la capacidad discriminativa fue de 0.8677, mostrando alta precisión para diferenciar entre los grupos de pre-frágil o frágil vs. robusto. Además, la prueba de bondad de ajuste de Hosmer-Lemeshow no fue significativa [χ²(8)=4,29; *p*=0,8301], lo que confirma que el modelo se ajusta adecuadamente a los datos observados. En este modelo, el índice MELD-Na se asoció significativamente con la fragilidad (OR=1,309; IC 95%: 1,05–1,63; *p*=0,017), al igual que el tiempo en lista de espera para trasplante hepático (OR=1,010; IC 95%: 1,00–1,02; *p*=0,042); sin embargo, la etiología de la enfermedad relacionada con el consumo de alcohol (OR=4,841; IC 95%: 0,48–48,83) no alcanzó significancia estadística (*p*=0,181). Un año después del trasplante hepático, el modelo mostró un pseudo R² de 0,1598, reflejando ajuste moderado, con AUC de 0,7630 que indica una capacidad discriminativa aceptable. La prueba de Hosmer-Lemeshow no fue estadísticamente significativa [χ²(8)=8,12; *p*=0,4220], respaldando así la validez del modelo; el índice MELD-Na mostró una asociación cercana a la significancia estadística (OR=1,237; IC 95%: 0,97–1,58; *p*=0,091), al igual que el tiempo en lista de espera (OR=1,006; IC 95%: 1,00–1,01; *p*=0,064), siendo la etiología relacionada con el consumo de alcohol la variable que presentó una asociación estadísticamente significativa con la fragilidad hepática (OR=4,694; IC 95%: 1,05–20.94; *p*=0,043) (Tabla 5).

**Tabla 5 -:** Variables que influyen en la fragilidad hepática al momento de inclusión en lista de espera para trasplante y tras el primer año postrasplante hepático. Análisis multivariante. Madrid, España, 2020-2021

**Variable**	**Inclusión en lista de espera para trasplante hepático**					**Un año después del trasplante hepático**				
	**Coeficiente de Regresión (β)**	**Error Estándar**	z*	***Odds Ratio* (Intervalo de Confianza del 95%)**	**Sig** ^†^ **(valor p)**	**Coeficiente de Regresión (β)**	**Error Estándar**	z*	***Odds Ratio* (Intervalo de Confianza del 95%)**	**Sig** ^†^ **(valor p)**
MELD-Na ^‡^	0,270	0,113	2,39	1,309 (1,050 – 1,633)	**0,017**	0,213	0,156	1,69	1,237 (0,966 – 1,584)	**0,043**
Tiempo en lista de espera (días)	0,011	0,005	2,03	1,010 (1,000 – 1,021)	**0,042**	0,007	0,003	1,85	1,006 (0,999 – 1,013)	0,064
EHRA ^§^	1,577	1,179	1,34	4,841 (0,480 – 48,832)	0,181	1,564	3,581	2,03	4,694 (1,052 – 20,941)	0,091
Constante	-3,634	1,673	-2,17	0,026 (0,001 – 0,701)	0,030	-2,518	0,110	-1,84	0,080 (0,005 – 1,183)	0,066

*z = Estadístico regresión logística; ^†^Sig (valor p) = Significancia estadística; ^‡^MELD-Na = *Model for End-Stage Liver Disease-Sodium*; ^§^EHRA = Enfermedad hepática relacionada con el alcohol

## Discusión

Los pacientes más frágiles son aquellos con descompensación avanzada de la función hepática, indicada por un alto índice MELD-Na. También son más frágiles los pacientes cuya enfermedad hepática está relacionada con el alcohol y que están desempleados o inactivos. Al año del TH, la fragilidad sigue relacionada significativamente con el índice MELD-Na y con la edad, siendo más frágiles los pacientes de mayor edad.

El LFI apoya al MELD-Na en la predicción de mortalidad en pacientes con disfunción hepática^(26)^. En nuestros resultados, el LFI mantiene esa correlación tanto antes como después del trasplante de manera directa y significativa. Al momento de inclusión en lista de espera, un mayor LFI también se relaciona significativamente con la EHRA. El alcohol y el síndrome metabólico empeoran la sarcopenia, afectando negativamente a los músculos y la señalización del crecimiento, y aumentando la resistencia a la insulina^(27)^. Las enfermeras de Hepatología deben educar a la población sobre las consecuencias de este problema en la enfermedad hepática, aconsejando sobre sus efectos y previniendo el consumo y el consumo abusivo^(28)^.

La relación entre fragilidad hepática, LFI, tiempo hasta el trasplante y cirrosis por consumo de alcohol en pacientes trasplantados es compleja y significativa. Los pacientes con cirrosis inducida por consumo de alcohol suelen presentar un mayor grado de fragilidad hepática, lo que puede influir en el tiempo de espera para ser trasplantados, pudiendo agravar su estado de salud general y los riesgos asociados al TH. La evaluación constante y el manejo adecuado del LFI son esenciales para mejorar los resultados en pacientes trasplantados con antecedentes de enfermedad hepática relacionada con el consumo de alcohol.

Nuestros resultados refuerzan la implementación de los objetivos de Desarrollo Sostenible de la Agenda 2030, siendo el objetivo principal *garantizar una vida sana y promover el bienestar de todos a todas las edades* y, en particular, el de *fortalecer la prevención y el tratamiento del abuso de sustancias adictivas y el consumo nocivo de alcohol* ^(29)^. Aunque nuestros resultados describen a la etiología relacionada con el alcohol como más frágil y nuestra muestra fue limitada, se mantiene la conclusión del estudio que propone que, aunque la fragilidad sea más común en pacientes con EHRA, se asocia con la mortalidad en lista de espera independientemente de la etiología de la cirrosis y debe aplicarse en todas las etiologías de esta patología^(30)^.

Aquellos pacientes inactivos, y sea por desempleo o por jubilación, son más frágiles. Una insuficiencia hepática deteriora el estado físico y cognitivo y empeora las condiciones físicas para poder desarrollar un empleo^(13)^. Sabemos que la fragilidad está relacionada con la sarcopenia, la malnutrición y la inactividad; pese a poder ser una consecuencia, también puede ser un factor agravante^(31)^, por lo que debemos poner el foco en la inactividad como riesgo de aumento de la propia fragilidad. La literatura describe la importancia de los programas de habilitación o pre-habilitación en pacientes cirróticos candidatos a trasplantes^(32-35)^. Para estos programas, la enfermera se postula como el profesional referente para evaluar la fragilidad, así como para la colaboración directa y estrecha con los profesionales de Atención Primaria y los familiares del paciente y su entorno, teniendo en cuenta su nivel socioeconómico y cultural^(7)^. Los cuidados enfermeros especializados en cirrosis o Hepatología se centran en nutrición, educación de los pacientes y cuidadores sobre posibles complicaciones, conciencia del riesgo de caídas y cuidado de la fragilidad de la piel, manteniendo un seguimiento periódico y revisión del cumplimiento de la medicación^(28)^. La Sociedad Estadounidense de Trasplantes propuso una evaluación multidimensional de la fragilidad en la práctica diaria incluyendo cuatro medidas: índice de Karnofsky, Actividades de la Vida Diaria (AVD), Índice de Fragilidad Hepática y prueba de los seis minutos marcha^(36)^. Las intervenciones para tratar la fragilidad hepática se deben centrar en lo siguiente: ejercicio físico, nutrición, tratamiento farmacológico y entrenamiento cognitivo^(37)^.

Tras el TH, los pacientes más frágiles tienen más riesgo de complicaciones e ingresos hospitalarios más prolongados^(11,38)^. Nuestros hallazgos no muestran diferencias significativas entre grupos de fragilidad, pero sí una tendencia similar a los resultados^(11)^. Después de un año tras el TH, el LFI únicamente se relaciona de manera directa con la edad, volviendo a su concepto primario de la Geriatría. Nuestros resultados también muestran que la fragilidad hepática sigue impactada por la etiología EHRA, el MELD-Na y el tiempo en lista de espera un año después del TH.

Tal como lo describe la literatura científica, el TH mejora la vida y la salud de los pacientes^(39-40)^. Nuestros resultados describen una mejora en las dimensiones físicas y mentales, en sintonía con los resultados de otros estudios. No obstante, debemos prestar atención al empeoramiento de los factores de riesgo cardiovascular^(39)^. En concordancia al tratamiento médico que se centra en alcanzar la dosis mínima de inmunodepresión que permita la viabilidad del injerto trasplantado previendo síndrome metabólico^(41)^, disfunción renal^(42)^ y cáncer^(43)^, desde Enfermería debemos poner el foco en el control y manejo de estos síntomas a partir de hábitos de salud modificables, dieta, ejercicio físico y prevención del consumo de sustancias tóxicas como tabaco y alcohol, manteniendo adherencia al tratamiento inmunosupresor^(44)^.

De entre los hallazgos secundarios de nuestro estudio, se destaca que la depresión se vincula directamente con la ansiedad y el MELD-Na durante el periodo en lista de espera, describiendo que aquellos pacientes con enfermedad hepática más avanzada y salud física empobrecida también sufren en su salud mental. Estos resultados ya se han descrito, concluyendo la necesidad de valorar la depresión en los pacientes frágiles^(37,45)^. Nuestros resultados se correlacionan indirectamente con la edad, describiendo que, al momento de inclusión en lista de espera, aquellos pacientes más jóvenes tienen puntuaciones de depresión más altas. Al año del TH, es la puntuación de ansiedad la que se correlaciona indirectamente con la edad. Nuestros resultados describen que la esfera de la salud mental en los pacientes más jóvenes se ve más afectada. Este impacto puede deberse a los miedos y las complicaciones de tener que someterse a un TH siendo joven y las consecuencias en su vida personal, impacto laboral y social y posible recurrencia de la enfermedad hepática^(46-48)^.

Las enfermeras deben centrar sus intervenciones en prevenir el alcoholismo y en la educación poblacional sobre sus consecuencias para la salud. También deben liderar programas de habilitación o prehabilitación física, fomentando el ejercicio físico dirigido en los pacientes frágiles para mejorar su calidad de vida y prevenir complicaciones tras el trasplante. Se debe evaluar la salud mental de los pacientes durante todo el proceso, focalizando en los pacientes más jóvenes. Los cuidados de Enfermería en pacientes trasplantados deben dirigirse al manejo de los factores de riesgo cardiovascular basándose en la modificación de hábitos como dieta, ejercicio físico y prevención del consumo de sustancias tóxicas como tabaco y alcohol.

Como fortaleza de nuestro estudio podemos mencionar el haber llevado cabo el seguimiento prospectivo utilizando índices e instrumentos de reciente implementación en unidades de TH. Las principales limitaciones serían el tamaño muestral, el haber sido desarrollado en un único centro, las pérdidas en la cumplimentación de la recogida de datos y la desestimación de los pacientes incluidos como trasplantes urgentes. Además, no se recogieron datos sociodemográficos al año del TH, lo que ha limitado el análisis de los resultados.

## Conclusión

El índice de fragilidad hepática describe el impacto físico de la enfermedad hepática y mejora tras el TH. Aquellos pacientes con enfermedad hepática avanzada, etiología relacionada con el alcohol y situación de desempleo son más frágiles durante el periodo pretrasplante. Después del TH, los factores de riesgo cardiovascular, diabetes e hipertensión arterial empeoran. La salud mental y la fragilidad de los pacientes mejoran tras el TH, siendo los más frágiles aquellos de mayor edad. La etiología relacionada con el consumo de alcohol, el MELD-Na y el tiempo en lista de espera impactan en la fragilidad durante todo el proceso del TH.
